# Effect of Marine‐Derived Scallop Peptide Hydrolysate on Immune Modulation and Gut Microbiota Restoration in Cyclophosphamide‐Induced Immunosuppressed Mice

**DOI:** 10.1002/fsn3.70421

**Published:** 2025-07-23

**Authors:** Muhammad Ilyas, Mujeeb Ur Rahman, Muhsin Ali, Ting Deng, Nabeel Ahmed Farooqui, Sharafat Ali, Hidayat Ullah, Yamina Alioui, Renzhen Ma, Shuming Lu, Liang Wang, Yi Xin

**Affiliations:** ^1^ Department of Biotechnology, College of Basic Medical Science Dalian Medical University Dalian China; ^2^ Department of Biochemistry and Molecular Biology, College of Basic Medical Science Dalian Medical University Dalian China; ^3^ Guangdong Provincial Key Laboratory of Research and Development of Natural Drugs, and School of Pharmacy Guangdong Medical University Dongguan China; ^4^ Stem Cell Clinical Research Center, National Joint Engineering Laboratory, Regenerative Medicine Center The First Affiliated Hospital of Dalian Medical University Dalian China; ^5^ Department of Gastroenterology First Affiliated Hospital of Dalian Medical University Dalian China

**Keywords:** apoptosis, cyclophosphamide (CTX), gut dysbiosis, microphages, scallop peptide hydrolysate (SCH), splenocyte

## Abstract

Cyclophosphamide (CTX), a chemotherapy agent, can weaken immune responses and disrupt gut microbiota, resulting in dysbiosis and inflammation. The gut microbiota, a composite community of microorganisms, plays a central role in regulating metabolism, supporting biological functions, and maintaining immune balance. Dysbiosis may lead to various systemic diseases and metabolic issues. This underscores the importance of immunomodulators to boost immune health and restore balance during CTX treatment. This study assessed the impact of scallop peptide hydrolysate (SCH), derived from scallops collected from a local seafood market in Dalian, China, on immune suppression, intestinal integrity, and gut microbiota composition in BALB/c mice with CTX‐induced immunosuppression. The findings demonstrated that SCH significantly restored intestinal mucosal integrity of goblet cells, enhanced the levels of tight‐junction proteins (Occludens, Zonula‐1, Mucin‐2, Claudin‐1), and improved the histological state of both the colon and spleen. SCH treatment resulted in increased body weight, improved immune organ indices, and enhanced levels of pro‐inflammatory cytokines and immunoglobulins, while reducing anti‐inflammatory cytokines. Furthermore, SCH exhibited no cytotoxic effects on RAW264.7 macrophages, promoting the formation of pseudopodia and enhancing phagocytic activity, thereby improving cell‐mediated immunity and splenic lymphocyte proliferation. Moreover, SCH therapy markedly increased the numbers of CD4^+^ and CD8^+^ T lymphocytes in the spleen, which CTX diminished. Additionally, SCH treatment decreased the Bacteroidetes/Firmicutes ratio and restored the biological balance of gut microbiota, increasing beneficial bacteria such as Muribaculaceae. These results indicate that SCH functions as a prebiotic and could serve as a functional food ingredient with potential therapeutic benefits for gastrointestinal disorders and immune regulation.

## Graphical abstract

Cyclophosphamide (CTX), a chemotherapy agent, can impair immune responses and disrupt gut microbiota, leading to dysbiosis and inflammation. This study evaluated the effects of scallop peptide hydrolysate (SCH) on immune suppression and gut microbiota in BALB/c mice treated with CTX. SCH significantly restored intestinal mucosal integrity, enhanced tight‐junction proteins, and improved colon and spleen histology. It increased body weight, immune organ indices, pro‐inflammatory cytokines (IL‐1β, TNF‐α), and immunoglobulins (IgM, IgG), while decreasing anti‐inflammatory cytokines (IL‐10, IL‐4). Additionally, SCH promoted macrophage activity, splenic lymphocyte proliferation, and increased CD4+ and CD8+ T lymphocytes, restoring gut microbiota balance by enhancing beneficial bacteria. These findings suggest that SCH functions as a prebiotic and may provide therapeutic benefits for gastrointestinal disorders and immune regulation.

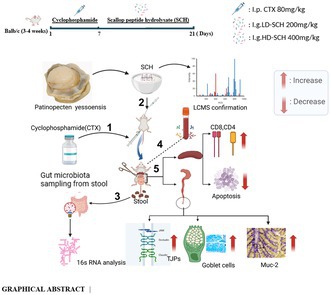

## 1 Introduction

The microbiome of the gut is a complex collection of billions of bacteria that inhabit a human body and serve various biological roles within the host's intestinal system (Fremont et al. 2013). Microbiota and the host share interactions in a microbial ecosystem within the gastrointestinal region, and the gut microbiota plays a crucial role in controlling host metabolism, such as breaking down carbohydrates to provide nutrients for the metabolic system's upkeep, vitamin synthesis, maintaining physiology, and preventing the invasion of pathogens by protecting the host from harmful microbes (De Santis et al. 2015; Chung et al. 2016). The four components of the intestinal barrier system—the mechanical, biological, chemical, and immunological barriers—cooperate to maintain homeostasis in the gastrointestinal tract (Zhang et al. 2020). Intestinal stem cells, endocrine cells, Goblet cells, absorptive cells, and Paneth cells comprise the many cell types that establish the mechanical barrier. These cells cooperate to preserve the integrity and functionality of the gut (Kurashima et al. 2013). A part of the chemical barriers, mucin‐2 (Muc‐2) is a goblet cell fluid that sticks to the epithelial layer to keep pathogens from assaulting the intestinal protective barriers (Hansson 2012; Kurashima et al. 2013). The microbiota promotes vascularization, villus thickening, epithelial junction maintenance, mucus production, cellular proliferation, and mucosal surface expansion in the intestine (Sommer and Bäckhed 2013; Kelly et al. 2015). Dysbiosis of the gut microbiome influences the development of the gut microenvironment and the emergence of metabolic syndromes and other systemic illnesses (Dwivedy and Aich 2011; Gamallat et al. 2019). However, commensal microorganisms are essential for food digestion, immunological response, and gastrointestinal secretions (Shi et al. 2017).

The gut microbiota has gained widespread recognition recently, and probiotics' sensible application as a functional food is well‐recognized (Delcenserie et al. 2008; Xiang et al. 2021). Infectious agents have a strong correlation with the onset or aggravation of disease and are known to cause infections. There is a known relationship between immunology, the gut microbiome, and the mucous membrane immunological system, even though commensal gut flora alters epigenetic and genetic factors and influences host health indirectly or directly (Kurashima et al. 2013; Gamallat et al. 2019). Disproportions in intestinal flora lead to downregulation of immunological response, affecting immunological system development and changing the systemic immune response in mucosal tissues (Dwivedy and Aich 2011; Shi et al. 2017). An aberrant immune system changes the nature of intestinal flora, which in turn affects the distant mucosa's immune response, resulting in an immune imbalance (Shi et al. 2017). An immunological response forms at the body's boundaries due to the host's reaction to the metabolites that microorganisms create, which changes the development of immune cells (Dwivedy and Aich 2011).

Considering its significant adverse impacts on folate and genetic material, cyclophosphamide (CTX) has been one of the most frequently administered pharmacological agents for the treatment of cancer and autoimmune disorders since 1958 (Zhang et al. 2020; Shams Ul Hassan et al. 2021). Besides tumor cells, a substantial dosage of CTX may lead to immune system downregulation, inflammation of the intestinal lining, disruption of gut microbiota, and heightened intestinal penetrability. Potential pathogens induce secondary infections in all cases (Hartmann and Meisel 2007; Prendergast and Jaffee 2007). Immunomodulators are essential for enhancing the immune response system to mitigate the detrimental impacts of CTX and to inhibit cytotoxicity and homeostatic imbalance (Kiewiet et al. 2018). CTX significantly impacts the immune system, particularly T cell populations. CD4^+^ T cells synchronize the immune defense response by activating B cells and CD8^+^ T cells, although CD8^+^ T cells directly eliminate cancerous or affected cells. CTX's immunosuppressive effects can reduce the function of both T cell types, compromising immune efficacy (Gasper et al. 2014). While a decrease in CD8^+^ T cell populations jeopardizes the body's capacity to eradicate tumors and virally contaminated cells, a decline in CD4^+^ T lymphocytes can affect the helper roles required for an efficient immune response (Mora‐Buch and Bromley 2021).

Macrophages are significant immune cells vital to the body's ability to carry out adaptive and innate immunological responses throughout illness and infection (Hwang et al. 2019). To increase immunological activity, the activated macrophages interact directly or indirectly with the immune system through various stimuli (Zhang et al. 2019). Activated macrophages activate these receptors by eliminating cell debris, foreign materials, and senescent cells and initiate intracellular signaling pathways. These pathways involve various molecular interactions, including inflammatory reactions that release inflammation‐promoting cytokines like tumor necrosis factor alpha (TNF‐α), Interleukin 1 Beta (IL‐1β), and Interleukin‐6 (IL‐6), and inflammatory agents like nitric oxide (NO) (Hwang et al. 2019). One commonly used chemotherapeutic medication for treating leukemia and tumors is CTX, an alkylating compound. As previously mentioned, the quick administration of CTX medication can lead to the disparity of T‐helper type 1 and type 2 (Th1, Th2) inclination and induce severe leukopenia, immunosuppression, and myelosuppression. Th1 and Th2 cells are essential to infection pathophysiology (Hwang et al. 2019). To generate cellular immunity against bacteria and different external microbes, interferon‐gamma stimulates Th1 cell differentiation (Romagnani 1992). Cytokine Interleukin‐10 (IL‐10) is an inflammation‐reducing cytokine that has been demonstrated to prevent Th1 cytokine secretion by preserving cytokine synthesis, differentiation, and proliferation (Elghazali et al. 1993; Couper et al. 2008). The past few years have seen studies on bioactive peptides, marine‐sourced protein hydrolysate, and functional meals for strengthening the immune system (Ambigaipalan and Shahidi 2017). Bioactive peptides are a set of polypeptides that are generated by enzymes during the digestion of natural and marine food proteins. The most popular technique involves hydrolyzing the whole protein with enzymes including pepsin, chymotrypsin, thermolysin, trypsin, alcalase, and pancreatin to create bioactive peptides (Dayakar et al. 2022). It physically consists of more than two to twenty amino acid residues; bioactive chemicals are more easily absorbed because of their tiny size and hydrophobic properties (Hartmann and Meisel 2007).

Consequently, it is believed that marine animals provide a source of novel physiologically active molecules for creating pharmaceuticals and crucial chemicals for nutritional health. Marine microorganisms' secondary metabolites are valuable for discovering natural antimicrobials with unique structures and potent activities, offering promising leads for novel antibiotic development (Pan et al. 2025). Recently, research has highlighted that compounds extracted from marine sources have demonstrated antibacterial activity against a range of pathogens (Hassan and Jin 2025). Particularly, marine peptides have become highly sought‐after due to their prospective to enhance well‐being and reduce the risk of disease (Ngo et al. 2012). Peptides may have a major impact on the intestines by controlling intestinal epithelium tight junctions, altering nutritional absorption, and regulating digestive enzymes (Shimizu 2004; Korhonen and Pihlanto 2006). Marine‐derived peptide hydrolysate is abundant in nutrients, safe, and advantageous to the body's systems. It has a range of uses, including enhancing immunity, decreasing cholesterol, raising blood pressure, combating cancer, and having antiviral, antibacterial, and anticancer properties (Ngo et al. 2012). According to reports, hydrolysate obtained from sharks protects intestinal epithelial cells and increases the generation of cytokines (Mallet et al. 2014). These protein derivatives have varying configurations and can consist of several or many amino acids. Low‐molecular‐weight peptides are safe, useful to the body physiologically, have good absorption, and offer several health benefits, including antibacterial, antihypertensive, antioxidant, antithrombotic, and anticancer properties. They also have immunomodulatory effects on both the adaptive and innate immune systems. Research indicates that bioactive peptides, which consist of short peptides, dipeptides, and tripeptides, are more rapidly absorbed and used in the digestive tract than amino acids with the same composition (Chen et al. 2019).

The scallop (Patinopecten yessoensis) is a giant shellfish inhabiting cold oceans. Because of its large adductor muscle, it has become a commercially significant marine culture species in northern China. After processing the scallop adductor muscle, the primary edible byproduct is the gonad, which has a high protein content (Han et al. 2019). The market is filled with various culinary products, and scallop consumption has recently surged. The majority of the diverse and abundant marine species are essential sources of food and medication for humans (Ambigaipalan and Shahidi 2017). According to earlier research, hydrolyses of scallop peptides may have various advantageous biological properties, such as antioxidant, anti‐inflammatory properties, and antihypertensive effects (Zheng et al. 2011). A recent study revealed that scallop male gonad hydrolysates had gelation properties, which may contribute to the development of marine‐derived proteins as a functional food base, including kamaboko gels, canned products, sausages, spreads, and delivery systems for bioactive chemicals (Yan et al. 2022). There has been a growing body of research on scallops' fundamental biology and immunology. Scallops depend on a comprehensive array of apoptosis, phagocytosis, and encapsulation of circulating hemocytes to eradicate invasive infections. They also synthesize immune effectors that target a broad spectrum of pathogens or respond to environmental stress (Song et al. 2015).

Earlier research found that collagen peptide could speed up the development of colitis by increasing the inflammatory response caused by dextran sulfate sodium (DSS). Intestinal microbial shifts, altered macrophage polarization, and elevated proinflammatory cytokines all play a role in this impact (Li et al. 2022). According to Khan et al. (2022), shrimp peptides have the potential to alleviate dysbiosis and alter the ecology of the gut microbiota in the intestines by decreasing the number of harmful bacteria at different taxonomic levels. Oyster peptides stimulate cytokines, modify gut microbiota, and ameliorate intestinal damage (Xiang et al. 2021). A recent study revealed that Octopus peptide hydrolysate may be utilized as an immunological component in healthy foods to control the immune system and shield the gut from inflammatory illnesses, according to a recent study (Ali et al. 2024). According to previous research, sea conch peptide hydrolysate is essential for restoring a healthy gut microbiome, which is defined as an imbalance between good and bad bacteria (Ullah et al. 2023).

However, the impact of scallop peptide hydrolysate (SCH) on mucosal integrity and gut microbiota has been studied less, and its functional activity remains undiscovered. According to our present comprehension, there is limited information on how SPH interacts with cyclophosphamide (CTX) in the context of immunosuppression. Therefore, this study investigates the potential of SPH to modulate the immunological response and restore intestinal flora in cyclophosphamide‐immunosuppressed mice. We hypothesize that SCH may aid in the treatment of immunosuppression by enhancing immunomodulation and promoting intestinal integrity.

## 2 Methods and Materials

Scallops (Patinopecten yessoensis) were obtained from the supermarket (Lvshunkou district, Loaning, Dalian, China). Papain was bought from Sigma‐Aldrich Trading (SAT) Co. Ltd. (Shanghai, China). Protein concentration assay kit (BCA) was bought from Jiancheng Bioengineering Institute (JBI), (Nanjing, China). ELISA kits for Immunoglobulin M (IgM), Interleukin 10 (IL‐10), Interleukin 4 (IL‐4), Tumor necrosis factor alpha (TNF‐α), Interleukin 1 Beta (IL‐1β), Immunoglobulin G (IgG), and rabbit antibodies were provided by Jiangsu Meibiao Biotechnology (JMB) Co. Ltd. (Jiangsu China); Apoptosis detection kit (Annexin V, FITC) was obtained from Dongren Chemical Technology (DCT) (Shanghai) Co. Ltd. All additional reagents utilized in the research were of laboratory grade.

### 2.1 Preparation of Scallop Peptide Hydrolysate (SCH) From Scallop

The hydrolysis process technique was applied to make scallop peptide SCH, as previously mentioned (Ullah et al. 2023). Grind the meat of scallops until they are entirely smooth, and then wash them in twice as much distilled water at a temperature range of 98°C for 60 min. The crushed scallop bits were combined with two liters of distilled water after passing them through 150 μm meshes. Next, 1% (w/w) of papain enzyme was added and incubated for 7 h at 50°C in a water bath under continuous agitation. After 7 h, the enzyme was deactivated at 100°C for 20 min; digested lysates were heated to 98°C for 15 min to terminate the enzyme activity. Lastly, the digested lysates were spun in a centrifuge at 12,000 rpm for 20 min to separate the supernatant. This was executed to make a hydrolysate from the scallops. The concentrations of SCH were determined using the Bradford technique.

### 2.2 Molar Mass Dissemination of Scallop Peptide Hydrolysate (SCH)

A matrix‐assisted laser desorption ionization time‐of‐flight mass spectrometry detector (Bruker, Germany) was used to analyze the molecular mass distribution of SPH.

### 2.3 Experiment Design and Animals

The Laboratory for Animal Studies and Health supplied twenty‐four female pathogen‐free BALB/c mice, ranging in age from 3 to 4 weeks, weighing 18 ± 2 g. The animal research could proceed by obtaining approval (Approval Code: 202410368) from Dalian Medical University's Experimental and Animal Ethics Committee. Mice were placed in conditions of 26°C ± 3°C temperature and 55% ± 5% humidity for each day, 10–12 h, and 10 h each day/night. Provided the mice with a regular diet and let them drink water whenever they wanted. After that, the mice were divided into four groups according to their weight after they had adjusted to their new environment for 1 week. The experimental methodology was detailed in previous work (Ullah et al. 2023). The control group, the Cyclophosphamide group (CTX), the high‐dose SPH group (HD‐SCH 400 mg/kg), and the low‐dose SPH group (LD‐SCH 200 mg/kg). Triads of mice were each given an intraperitoneal injection of cyclophosphamide at an 80 mg/kg/day dosage for 7 days, adjusted for mice's total weight. Control and SCH groups received an identical volume of normal saline. From day 8 to day 21, mice in the two groups received 200 or 400 mg/kg of SCH orally via gavage, depending on their body weight. Mice in the Control and CTX groups were administered an equivalent volume of distilled water orally through gavage.

### 2.4 Immune Organ, Body Mass Index, Water, and Food Index Measurement

Daily measurements of weight were taken throughout the administration period. After the last intragastric dose, mice were slaughtered 24 h later. Every 3 days, the amount of food and water consumed was recorded. Then, each mouse's spleen, liver, thymus, and colon were carefully removed and weighed in an aseptic manner. The following formula was used to compute the colon, thymus, and spleen indices:

$$\text{Thymus or Spleen index}\;\left(\text{milligram}/g\right)=\text{Organ weight}\;\left(\mathrm{mg}\right)/\text{mice weight}\;\left(g\right)$$

### 2.5 Immunoglobulin and Cytokine Serum Analysis via ELISA

The mice were euthanized the preceding day, and their blood was obtained by excising their eyes in a sterile environment. To extract serum, blood samples were spun at 4°C for 10 min at 3500 rpm. The 1.5 mL tube was used to collect the serum, which was then kept at −20°C. The ELISA kit instructions were followed to measure the amounts of immunoglobulin (IgG and IgM) and cytokines TNF‐α, IL‐10, IL‐1β, and IL‐4 in the serum.

### 2.6 Determination of Intestinal mRNA

The mRNA expression levels of IL‐10, TNF‐α, TGF‐β, and IL‐4 were quantified. The whole RNA was isolated from colonic tissue by the producer's process, utilizing the Vazyme RNA Extraction Kit (Vazyme Biotech Co. Ltd.). The entire RNA was preserved at 60°C–80°C. Complementary DNA was subsequently transcribed using the HiScript II Q RT SuperMix (Vazyme Biotech Co. Ltd). The ChamQ SYBR green qPCR MasterMix kit performed quantitative PCR utilizing Bioer LightGene 9600 analyzers (Hi‐tech (Binjiang) District, Hangzhou, 310053, China). Table 1 displays primer sequences. The rt. PCR was performed at 95°C for 5 min, followed by 35 cycles of 95°C for 20 s, primer annealing at 60°C for 30 s, and extension at 72°C for 30 s. Each sample was subjected to three tests, employing the Gene 9660 program for calculating and analyzing relative expression, while GraphPad Prism 10.2.3 was utilized to evaluate intergroup differences.

**TABLE 1:** PCR primers list used for qPCR.

Gene	Forward (5′–3′)	Reverse (3′–5′)
*IL‐4*	ACAGGAGAAGGGACGCCAT	GAAGCCCTACAGACGAGCT
*TNF‐α*	CATCTTCTCAAAATTCGAGTGACAA	TGGGAGTAGACAAGGTACAACCC
*IL‐10*	CGGGAAGACAATAACTGCACCC	CGGTTAGCAGTATGTTGTCCAGC
*TGF‐β*	CATTGCTGGTCCAGTCTGCTTCG	TGGTGAATGACAGTGCGGTTATGG
*β‐actin*	ATCGCTGCGCTGGTCG	GTCCTTCTGACCCATTCCC

### 2.7 Quantification of Splenocyte Proliferation

One hundred microliters of spleen cellular suspension was applied to 96‐well cell culture plates. Each experimental and control group completed three repetitions, both with and without Con A (7.5 μg/mL). After 72 h of incubation at 37°C with 5% CO_2_, 200 μL of a 5 mg/mL solution of 3‐(4,5‐dimethylthiazol‐2‐yl)‐2,5‐diphenyltetrazolium (MTT) was added to each well. The same conditions were employed to incubate the plates again for 4 h. Meticulously remove the supernatant following centrifugation of the plates at 250 g for 15 min at 4°C post‐incubation. The 96‐well plate was incubated for 10 min after the addition of 150 μL of DMSO (dimethyl sulfoxide) to every well. A microplate reader measured optical density (OD) at 570 nm. The formula for computing the stimulation index (SI) is as follows:

SI=ODvalue withConA/ODvalue withoutConA

### 2.8 Measurement of Splenocyte Apoptosis

One milliliter of suspension of splenocytes was spun at 1000 g for 10 min at 4°C. After the supernatant was discarded, 200 μL of binding buffer was added to the cells. Subsequently, 5 μL of Annexin V‐fluorescein FITC were amalgamated with the remaining components and incubated at normal temperature for 10 min. Subsequently, a staining solution comprising propidium iodide (PI) was introduced and meticulously blended in a dark setting before application. The NovoExpress flow cytometer was employed to assess Annexin V‐F fluorescence on the green channel and PI fluorescence on the red channel.

### 2.9 Analyzing Spleen for CD8 ^+^ and CD4 ^+^ T Lymphocytes

Spleen lymphocytes were isolated, resuspended in PBS, and calculated to a concentration of 1 × 106/mL. Cell suspensions of 100 μL were treated with 0.5 μL of anti‐CD4^+^ (FITC) and 1.25 μL of anti‐CD8^+^ (APC) for 30 min at 4°C in the dark. PBS was used to wash the spleen lymphocytes twice. The ratio of CD8^+^ and CD4^+^ T cell subsets was assessed utilizing a NovoExpress flow cytometer.

### 2.10 Assessment of Morphology of RAW264.7 Murine Macrophages

Neutral red phagocytosis was investigated using the method described previously (Wang et al. 2010). Cells were seeded at a density of 1 × 10^5^ cells/mL in 2 mL on a 6‐well cell culture dish once the cells reached approximately 80% confluency. After incubation overnight, the cells were administered different amounts of SCH (50, 100, 200, and 400 μg/mL). The positive standard group received 1 microgram/mL of lipopolysaccharide (LPS), while the opposing group was kept in comprehensive growth media. All treatments of RAW264.7 cells were performed for 24 h in a humidified incubator with 5% CO_2_ at 37°C. The morphology of the RAW264.7 cells was examined and documented using a light microscope.

### 2.11 Histopathological Examination of Spleen and Colon

The spleen and colon were taken out, preserved in 10% formalin, and subsequently processed. Slim slices of 5 were prepared using a stained microtome with hematoxylin and eosin (HE) staining technique, following (Fischer et al. 2008). Histological alterations were found during microscopy. Periodic acid staining was utilized to evaluate the mucous cells and the width of the mucous colonic epithelial tissue. The slides were deparaffinized with xylene and subsequently rehydrated using a series of ethanol dilutions. Subsequently, the slide was subjected to periodic acid treatment for 5 min at ambient temperature. The slides were rinsed three times with ultra‐purified water, using a 6:2 ratio. The Schiff solution was subsequently added to slides for 8–10 min at ambient conditions; after that, the slides were enclosed in a box and subjected to 6 min of washing under tap water. A secondary stain of hematoxylin was administered and rinsed with running tap water for 7 min, followed by dehydration using ethanol and clarification with xylene. Neutral balsam (solar bio) cat‐G8590 was used to cover the slides. An impartial researcher assessed histological alterations in a blinded fashion.

Immunohistochemistry was used to assess the expression of Mucin‐2 in colonic tissue. Subsequently, 5 μm sections of paraffin‐embedded colon tissue were sliced, positioned onto a positively charged surface, and paraffin removed using xylene. Revitalize in a succession of ethanol, adhering to the technique of immunohistochemical (IHC) staining kits SP‐9001 (Zhongshan Goldenbridge Biotechnology, Beijing, China) as per the manufacturer's instructions. The results were assessed in a semiquantitative manner, with each slide randomly examined for immunostained cells across 5 domains for 3 instances.

Immunofluorescent labelling was employed to assess the protein expression of Occludin, Zonula Occludin (ZO‐1), and Claudin‐1 in the colon. Subsequently, 5 μm embedded in paraffin colon tissue was sectioned and positioned onto a positively charged slide, using xylene for deparaffinization and ethanol gradient for rehydration through a sequential process. Tissue slices underwent treatment in citrate buffer solution for 30 min and antigen retrieval buffer at 100 watts in a microwave, followed by a cooling period of 1 h. Tissue slides were subsequently incubated in a 3% BSA‐blocking solution for 1 h. Tissue slides were treated overnight at 4°C with ZO‐1, Claudin‐1, and Occludin antibodies at a dilution of 1:200. Tissue slides were treated with conjugated secondary antibodies (Alexa 488) for 60 min following washing, and DAPI was employed for nuclear staining. Images were acquired via a confocal scanning microscope.

### 2.12 Microbiota and Stool DNA 16S rRNA Pyrosequencing

The PowerMax (stool) DNA isolation kit (MoBio Laboratories, Carlsbad, CA, USA) was utilized for the extraction of total fecal microbial genomic DNA from all group mice, which were preserved at −20°C. The quantity and quality of DNA were assessed via a spectrophotometer (OD). PCR amplification of the microbial 16S rRNA gene V4 region was conducted utilizing the primer forward 341f (CCTACGGGAGGCAGCAG) and the reverse 518r (ATTACGCGGCTGCTGG), followed by sequencing on the NovaSeq6000 Illumina platform at GUHE, Info Technology Co. Ltd. (Hangzhou, China). The QIIME tool was utilized to evaluate the quantity and variety of distinct bacterial populations in samples, employing alpha diversity criteria like the Shannon and richness indices (Caporaso et al. 2010). In contrast, beta diversity was represented using a weighted UniFrac distance employing principal coordinate analysis and nonmetric multidimensional scaling (NMDS). FAPROTAX and insect databases were utilized to investigate ecologically significant compounds and their functions in bacterial clades.

### 2.13 Statistical Analysis

Each value was presented as the mean and the standard error of the mean. Statistical data analysis was performed using GraphPad Prism version 10.2.3. To assess significance, a one‐way analysis of variance was utilized, followed by Tukey's various comparison tests. R and QIME software were applied for 16s RNA data analysis. Additionally, several bacterial classes, biologically significant functions, and notable phenotypes were examined using the STAMP package v2.1.3 (Parks et al. 2014), FAPROTOAX (Louca et al. 2016), and Bugbase tools for the statistical evaluation of genomic data (Thomas et al. 2016).

## 3 Results

### 3.1 SCH‐Related Proteins Concentration

Figure S1 shows the maximal protein concentration and the hourly increase in SCH concentration when treated with 1% papain. SCH analysis using SDS gel electrophoresis demonstrated that these proteins are peptides with relatively small molecular weights, mostly under ten kDa (Khan et al. 2022).

### 3.2 Distribution of Molecular Mass in Scallop Peptide Hydrolysates

**FIGURE 1:**
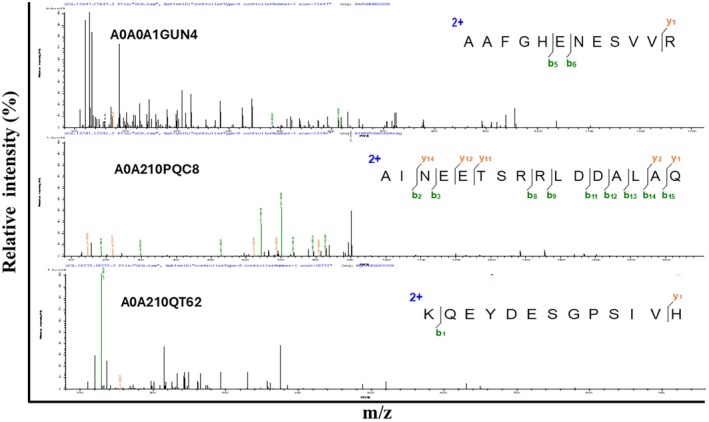
Scallop bioactive peptides in the hydrolysate were analyzed using mass spectrometry. The generated peaks represented the peptide samples with variable molecular masses and varying amounts of various amino acids.

Examine the SCH following enzymatic breakdown by papain. For mass spectrometry analysis, the SPH was freeze‐dried. As shown in Figure 1, along with Figure S1 and Table 1, the mass spectrometry data reveal three peaks in Figure 1 and an additional nine peaks in the Supporting Information. These peaks correspond to various peptides with distinct combinations of amino acids and varying molecular weights (Table 2).

**TABLE 2:** The main peptides in scallop hydrolysate differ in their amino acid composition and molecular mass.

Serial no	Molecular weight (kDa)	Protein accession number	Length	Identified peptides	Charge
1	1799.876	P04113	15	YIKDLLENMGDNFTK	2;3
2	1757.762	A0A210Q4N	13	NWDDMEKIWHHTF	2;3;4
3	1713.767	P05963	15	EAFSMLDVDRDGFVN	2
4	651.75	A0A210PDP3	14	TDLQEDLEGNVKYE	2
5	1237.812	A0A0A1GUN4	11	NGIILNKLLIK	2
6	1200.61	A0A210PR34	10	AIRNDEELNK	2;3
7	1344.652	A0A210QT62	11	EKEADDLEQLR	2;3
8	1600.806	A0A210QV72	13	EKEADDLEQLRQK	3
9	1928.948	A0A210QDC3	18	DEGGFAPNILENKEGLNL	2
10	1361.668	A0A210QVY2	12	NMLDKVNEMIVG	2
11	1463.748	A0A210QSN4	13	KEAPGPLNFTMFL	2
12	1308.631	A0A210PQC8	11	LDIDSEPHEVR	3

### 3.3 Scallop Peptide Hydrolysate Ameliorative Effect on Immunosuppressed BALB/c Mice

**FIGURE 2:**
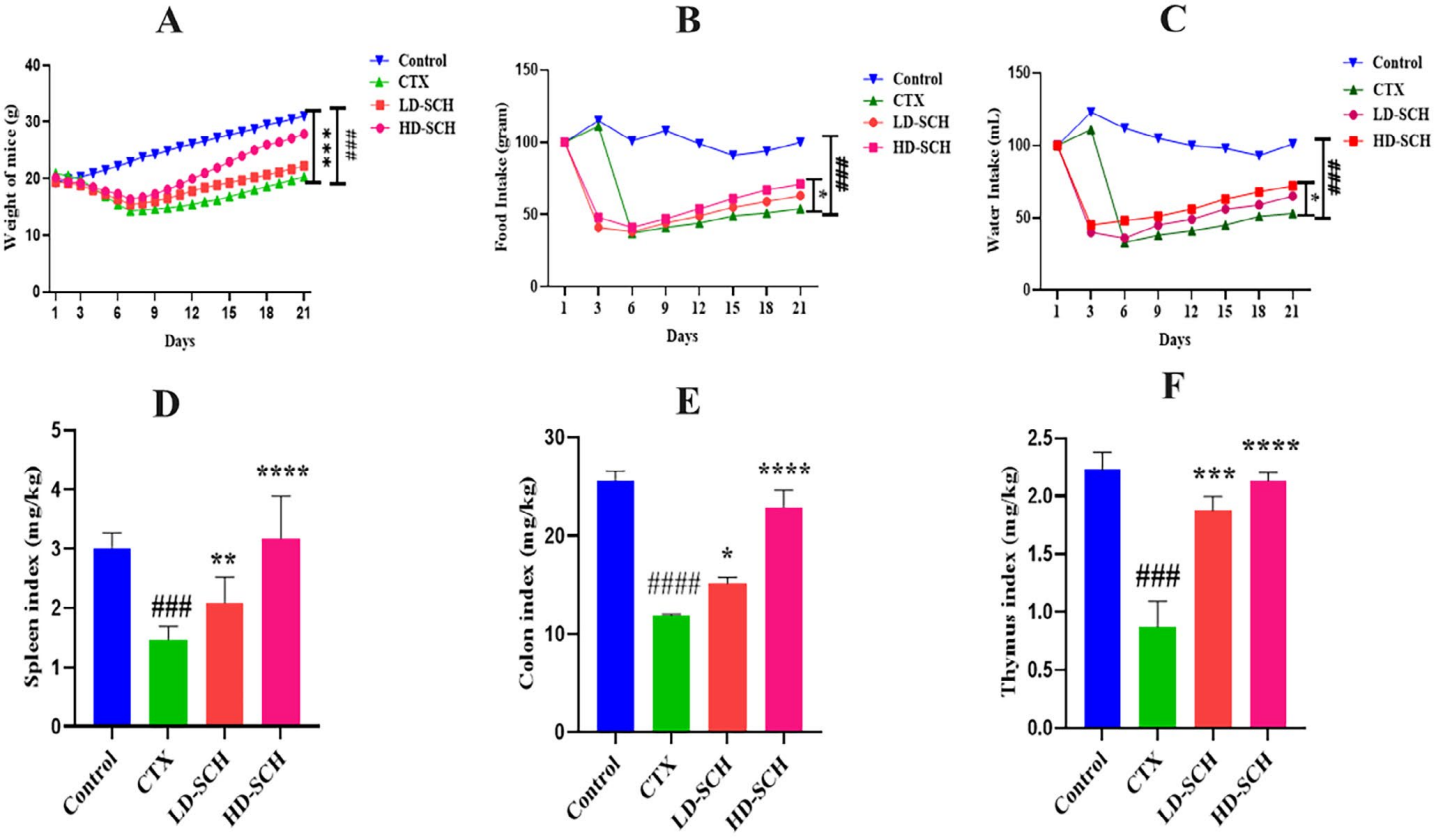
SCH affects health metrics. (A) Weights of mice, (B) Food intake, (C) Water intake, (D) Spleen index, (E) Colon index, and (F) Thymus index, Control (normal saline injection and normal saline gavage), CTX (80 mg/kg/day for 7 days followed by normal saline), HD‐SCH (400 mg/kg from day 8–21), and LD‐SCH (200 mg/kg from day 8–21). The mean ± SD (*n* = 6) is used to express the data. Control *p < 0.05, *p < 0.05, **p < 0.01, ***p < 0.001, ****p < 0.0001, ^###^ *p* < 0.001, ^####^ *p* < 0.0001 comparison to the CTX group.

All mice were examined following CTX administration, and daily fluctuation in body weight was recorded. The CTX group's body weight was significantly less than that of the control group (*p* < 0.001), Figure 2A. Consumption of CTX also influenced the CTX group's consumption of food and drink, as illustrated in Figure 2B,C. On the other hand, food and water intake increased due to SCH treatment. Additionally, the colon length of the CTX group was smaller than that of the control group (*p* < 0.0001). The spleen and thymus organ indexes were increased in the SCH treatment group compared to the CTX group. The immune organs index showed a statistically significant difference (*p* < 0.05) and (*p* < 0.001) in the groups receiving high and low SCH doses. At the same time, there were no such distinctions in the control group. Consuming CTX results in inflammation, which causes the colon, thymus, and spleen to enlarge and raises indices compared to a control. Conversely, the SCH group's indices were comparable to the control group's Figure 2D–F.

### 3.4 SCH Increased Serum Cytokine and Immunoglobulin Levels

**FIGURE 3:**
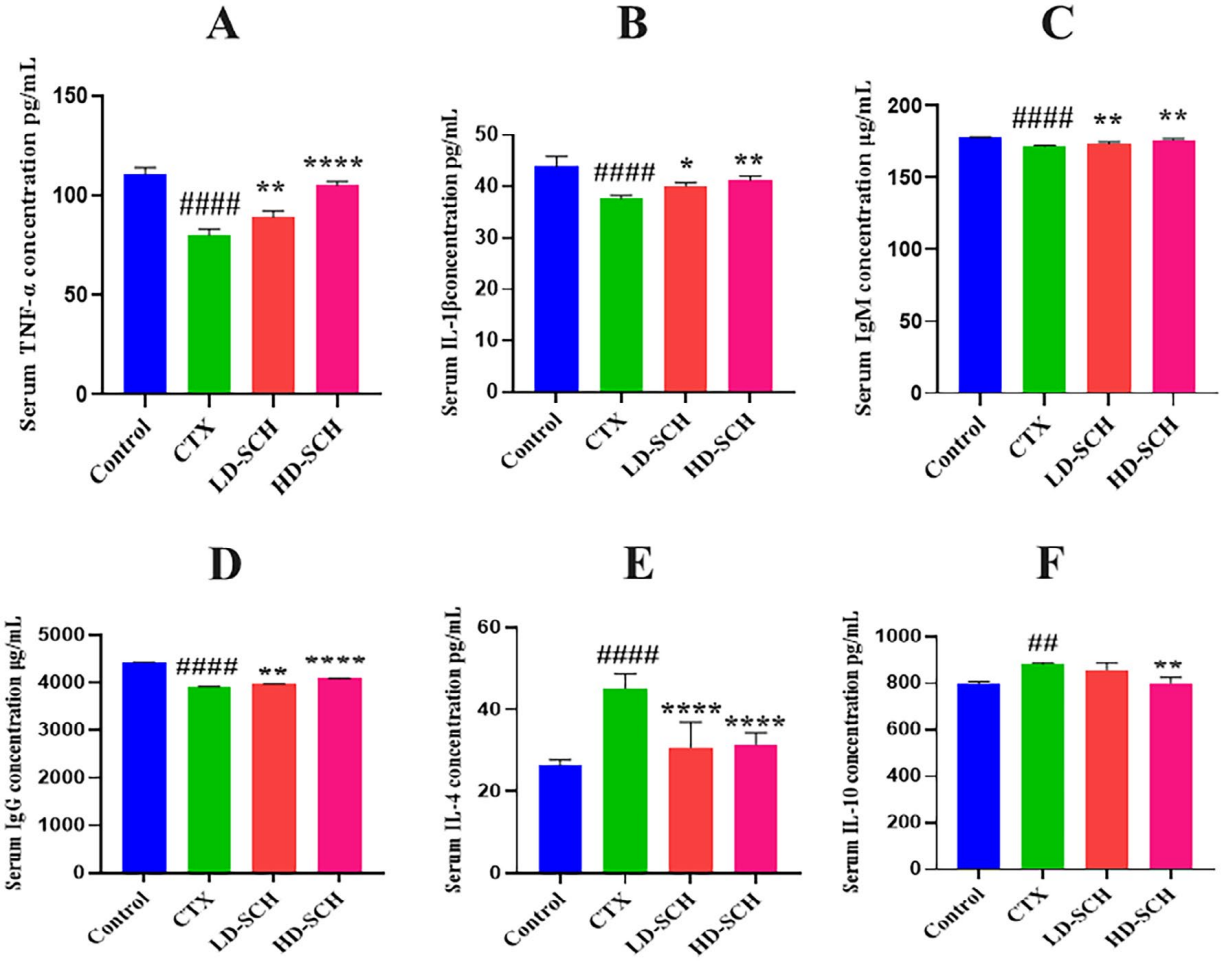
Impact of SCH on serum immunoglobulin levels and cytokine concentrations. (A) IL‐1β, (B) TNF‐α, (C) IgM, (D) IgG, (E) IL‐4, (F) IL‐10 were measured by ELISA, followed by SCH treatment groups. Data presented as mean *±* SEM. ^####^ *p* < 0.0001, ^##^ *p* < 0.01, comparison to Control *p < 0.05. *p < 0.05, **p < 0.01, ****p < 0.0001 comparison to CTX group.

**FIGURE 4:**
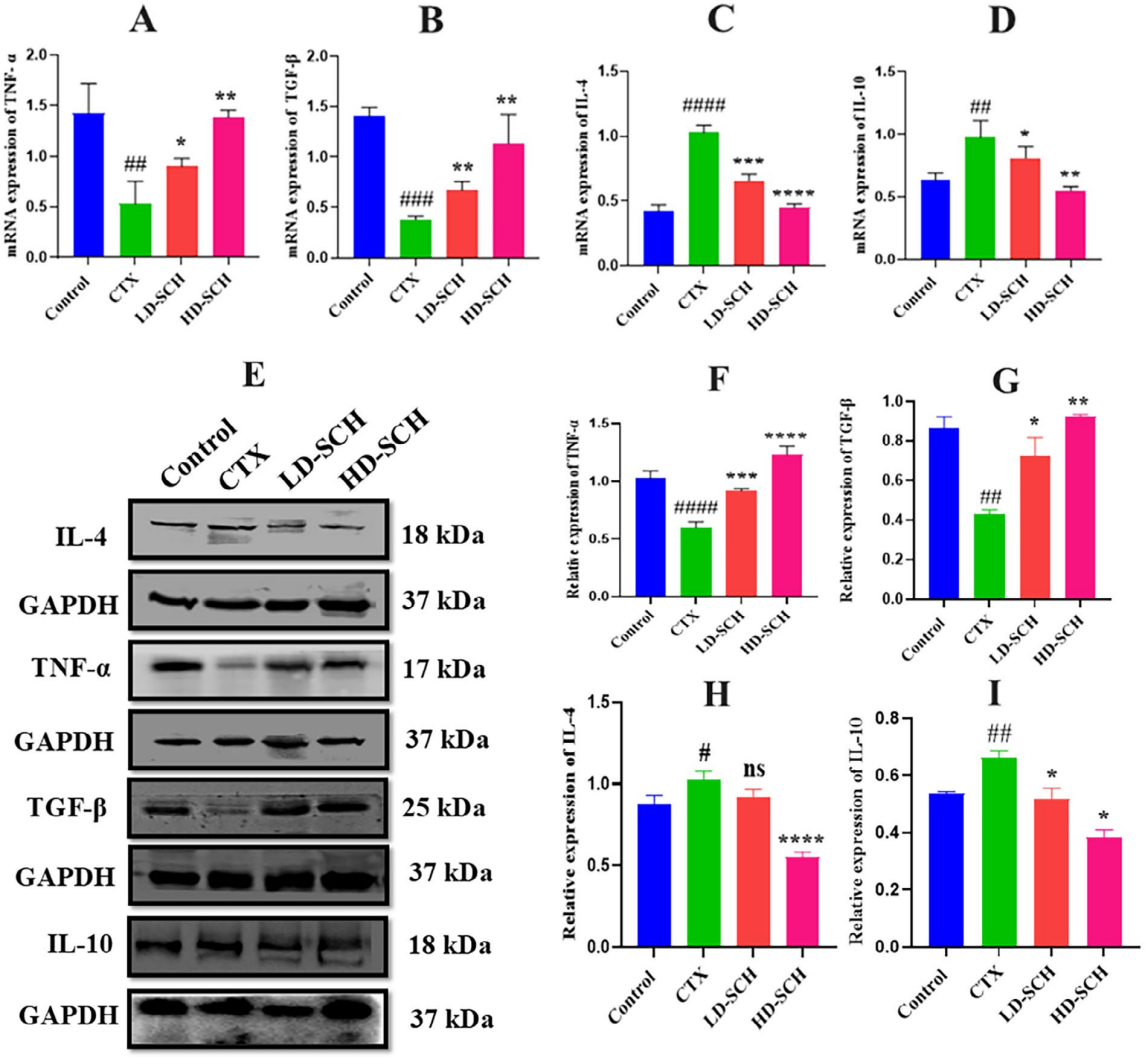
Relative mRNA and protein expression in colonic tissue. (A) TNF‐α (B) TGF‐β, (C) IL‐4, (D) IL‐10. mRNA levels and Western blot analysis of IL‐4, TNF‐α, TGF‐β, and IL‐10 (E–I) in colon tissue were normalized with GAPDH, and results are shown as mean *±* SD. ^#^ *p* < 0.05, ^##^ *p* < 0.01, ^###^ *p* < 0.001, ^####^ *p* < 0.0001 CTX group, *p < 0.05, **p < 0.01, ***p < 0.001, ****p < 0.0001 compared with the control group.

There was a considerable decrease in the cytokine concentration of IL‐1β and TNF‐α Figure 3A,B. In contrast to the control group, all SCH treatment groups demonstrated a significant increase (*p* < 0.05, *p* < 0.01, *p* < 0.0001) in serum IgM and IgG concentrations Figure 4C,D. Similarly, CTX immunosuppressed mice demonstrated a significant increase (*p* < 0.05, *p* < 0.01, *p* < 0.0001) in IL‐10 and IL‐4 concentrations Figure 4E,F relative to the control group. While LD‐SCH promoted immunoglobulin and cytokines, HD‐SCH was significantly more effective.

### 3.5 SCH Treatment Regulates Cytokine mRNA and Protein Expression in the Colon

To validate the findings, the mRNA expression levels of various cytokines (both anti‐inflammatory and pro‐inflammatory) in the colon were examined. The results indicated a significant reduction in pro‐inflammatory cytokines, specifically TNF‐α (*p* < 0.05) and TGF‐β (*p* < 0.01), in the CTX group relative to the control group, as illustrated in Figure 4A,B. The mRNA expression levels of the anti‐inflammatory cytokines IL‐4 (*p* < 0.0001) and IL‐10 (*p* < 0.01) were significantly elevated in the CTX group Figure 4C,D. Conversely, the SCH treatment groups, particularly the high dose, demonstrated a marked elevation in mRNA expression of TNF‐α (*p* < 0.05) and TGF‐β (*p* < 0.05), alongside a decrease in mRNA expression of the anti‐inflammatory cytokines IL‐10 (*p* < 0.01) and IL‐4 (*p* < 0.0001) relative to the CTX group Figure 4C,D. Additionally, Western blot demonstrates the relative protein expression of TNF‐α, TGF‐β and IL‐4, IL‐10 across the treatment groups, with GAPDH as a loading control. Bands corresponding to IL‐4, IL‐10 TNF‐α and TGF‐β indicate protein levels, with significant variations among groups, as demonstrated in Figure 4E. The CTX group shows decreased TNF‐α (*p* < 0.0001) and TGF‐β (*p* < 0.01) expression compared to the control group. Both Low‐ and high‐dose SCH groups exhibit significantly increased TNF‐α levels, especially the HD‐SCH group, suggesting a dose‐dependent effect of SCH treatment Figure 4B. Furthermore, in Figure 4C, the expression of IL‐4 and IL‐10 CTX treatment increased expression as compared to the control group, while LD‐SCH shows a recovery effect. Notably, the HD‐SCH group displays a pronounced decrease in IL‐4 (*p* < 0.0001) and IL‐10 (*p* < 0.01) compared to the CTX group, indicating enhanced anti‐inflammatory responses with higher dosing. These findings indicate that cyclophosphamide induces an inflammatory response with altered levels of TNF‐α, TGF‐β, IL‐4, and IL‐10. Treatment with SCH, particularly at higher doses, effectively modulates these cytokine levels, suggesting a potential therapeutic role in mitigating cyclophosphamide‐induced immunosuppression.

### 3.6 SCH Impact Splenic Lymphocytes of CD8 ^+^ and CD4 ^+^ T Cells

**FIGURE 5:**
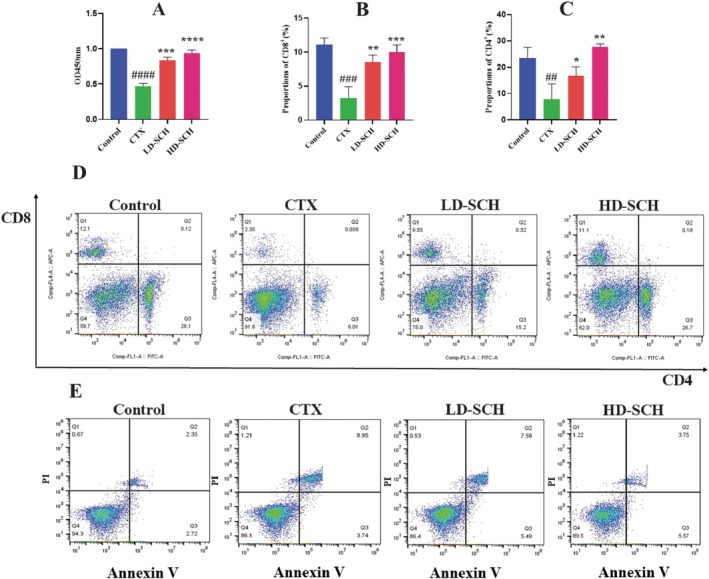
Influence of SCH on proliferation of splenic lymphocytes inspired by LPS upon T‐lymphocyte subsets and effects on splenic lymphocyte apoptosis. (A) The proliferation of splenic lymphocytes is generated by LPS. (B, C) Representative results of the CD8 and^+^ CD4^+^ subsets of the flow cytometry analysis. (D) Representative images of the splenic T cell subsets. (E) The impact of SCH on the demise of lymphocytes in the spleen. Each quadrant represents a different type of cell death: Necrotic, late apoptotic, normal, and early apoptotic. Propidium iodide, or P. I. Standard, given a solution of saline; cyclophosphamide (CTX) given intraperitoneally; cyclophosphamide (SCH) given intraperitoneally first and then SCH dose‐dependently administered. Data are expressed as mean ± SD (*n* = 6). *p < 0.05, **p < 0.01, ***p < 0.001, ****p < 0.0001 vs. CTX group, ^##^ *p* < 0.01, ^###^ *p* < 0.001, ^####^ *p* < 0.0001 compared with the control group.

Lipopolysaccharide‐stimulated cell proliferation was assessed after splenic lymphocytes were extracted from several mouse groups. Compared to the control group, the CTX group's LPS‐induced splenic lymphocyte proliferation was much lower, as seen in Figure 5A. However, following HD‐SCH treatment, this proliferation further increased. Mice with immunosuppression used flow cytometry to measure the quantity of CD8^+^ and CD4^+^ T cells in the spleen. Compared to the control group, the CTX group's CD4^+^ and CD8^+^ T lymphocyte percentages were noticeably lower. However, in SCH groups, the decrease in CD8^+^ and CD4^+^ T cells was followed by an increase, suggesting that SCH had an impact on immunological enhancement, which was uniformly shown in the HD‐SCH group Figure 5B–D.

### 3.7 SCH Impact on Splenic Lymphocyte Apoptosis

Scallop peptide hydrolysate influences splenic lymphocyte apoptosis. Compared to the control group, there was a substantial increase in the early, late, and overall splenic lymphocyte apoptotic rate (*p* < 01). The early, late, and overall apoptosis rates were considerably reduced in the HD‐SCH group compared to the CTX group (*p* < 0.01), as depicted in Table 3 and Figure 5E.

**TABLE 3:** Impact of SCH on the apoptotic rate of splenic lymphocytes.

Group	Early apoptosis %	Late apoptosis %	Total
Control	2.70 ± 0.08	2.32 ± 0.018	5.03 ± 0.27
CTX	5.61 ± 0.941	8.92 ± 0.012	14.54 ± 0.92
LD‐SCH	5.28 ± 0.112	7.54 ± 0.022	12.83 ± 0.13
HD‐SCH	4.59 ± 0.490	3.740 ± 0.015	8.33 ± 0.49

### 3.8 Cytotoxicity of SCH on RAW264.7 Cells

**FIGURE 6:**
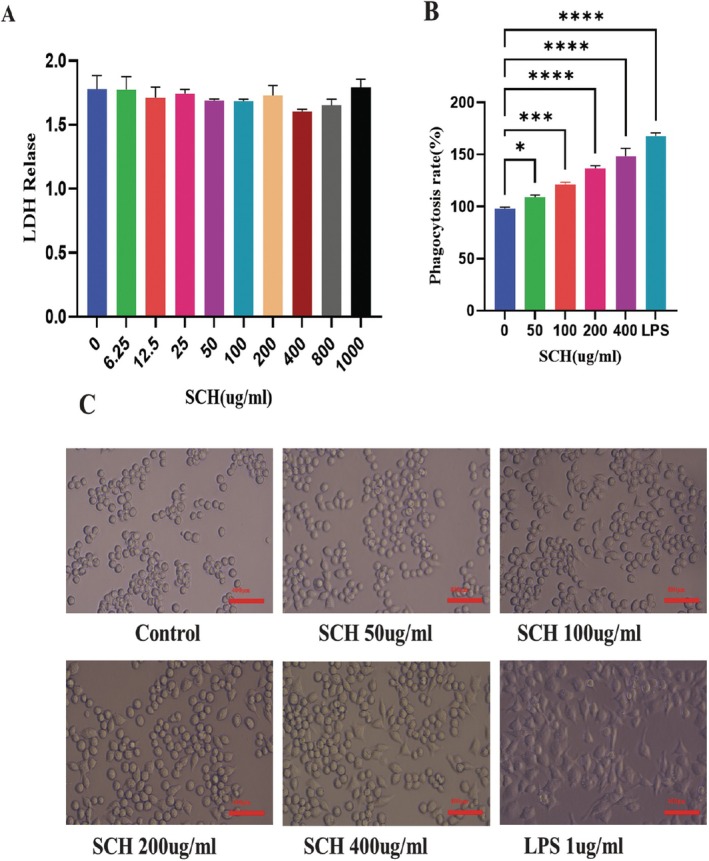
SCH treatment effects, lactate secretion, RAW264.7 cell morphological assessment, and phagocytosis rate. (A) SCH treatment and lactate dehydrogenase (LDH). (B) RAW264.7 cells at different concentrations for 24 h. (C) Morphology of RAW264.7 cells at different concentrations of SCH and LPS treatment (magnification 40×). ‘*’ represent *p* < 0.05, ***p < 0.001, and ****p < 0.0001.

Compared to cells administered with 0 μg/mL SCH, RAW264.7 cells administered with SCH at concentrations ranging from 6.25–1000 μg/mL did not exhibit any notable cytotoxic impact or LDH release Figure 6A.

### 3.9 Analysis of RAW264.7 Cell Morphology and the Impact of SCH on Phagocytosis

As seen in Figure 6B, the RAW 264.7 macrophage cells undergo morphological alterations. The cells were subjected to treatment with SCH or LPS under an inverted microscope. Macrophage morphology was typical, with average roundness and smoothness in unstimulated cells. In contrast, LPS‐stimulated macrophage cells stretched out and formed many pseudopods; they also had uneven, polygonal, and rough forms. Macrophage cells treated with SCH exhibited two distinct populations: one with expanded cell size and shape and another with irregular and circular forms, as well as pseudopodia. As the amount of administered SCH increased, the cells spread, and the production of pseudopodia became prolonged.

The neutral red assay was employed to assess the phagocytic activities of RAW264.7 cells. The phagocytosis rates of the SCH are contingent upon dosage in comparison to the control group. Compared to the control group, the groups receiving 50–400 μg/mL SCH had an increased rate of neutral red absorption by macrophages. In contrast to the SCH treatment group, the LPS group showed a marginally higher rate of phagocytosis for neutral red internalization. The data indicated that RAW264.7 macrophage cells have improved nonspecific immunity and phagocytic capability at varying SCH concentrations Figure 6C.

### 3.10 SCH Improves Mouse Colon, Spleen Histomorphology, and Goblet Cell Production

**FIGURE 7:**
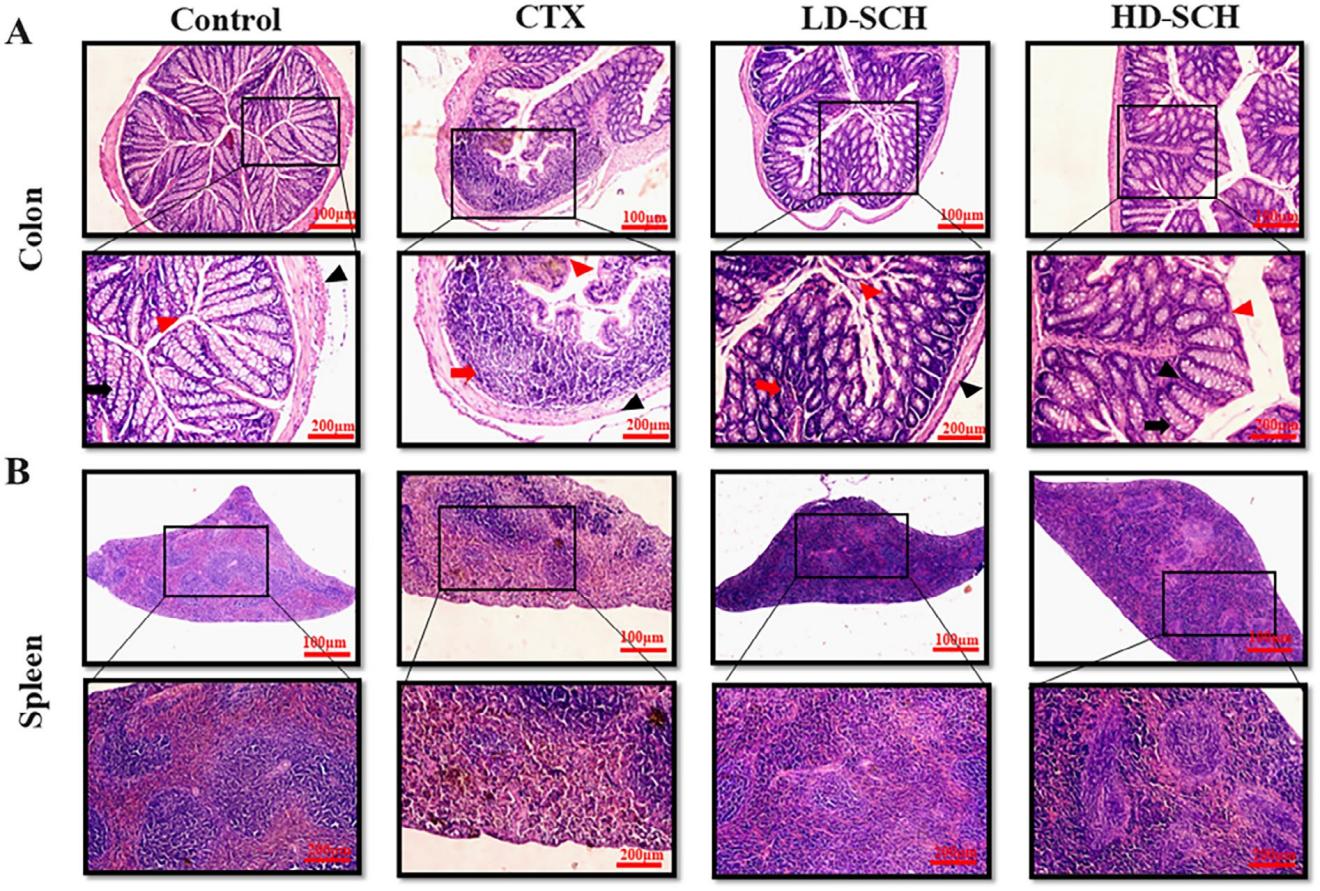
Changes in colon and spleen tissue morphology in mice treated with CTX and the impact of SCH on these organs (A) Colon (B) Spleen. Staining with H&E (upper 10*×*) (lower 20*×*) Control, given with saline; CTX, Cyclophosphamide given intraperitoneally; SCH, Cyclophosphamide given intraperitoneally first, then SCH treatment groups. Magnification: 20× scale bar 100 μm.

The pathological examination using HE staining revealed that the colon histomorphology indicates enhanced penetrability following CTX administration, characterized by villi rupture, a reduction in goblet cell elongation, and superficial crypts, as illustrated in Figure 7A. The proportion of goblet cells in the colon was assessed following cyclophosphamide treatment. The pathological examination of the CTX group's spleens showed white pulp atrophy, bleeding, and necrosis; also, there was a mixture of red pulp and white pulp in Figure 7B spleen. In contrast, the pathological modifications in the colon and spleen that CTX caused were much reduced after SCH treatment, and the changes observed in the CTX group were eliminated. In addition, results from histopathology demonstrated that HD‐SCH repaired CTX‐induced damage to the colon and spleen in a dosage‐dependent way Figure 7A,B.

**FIGURE 8:**
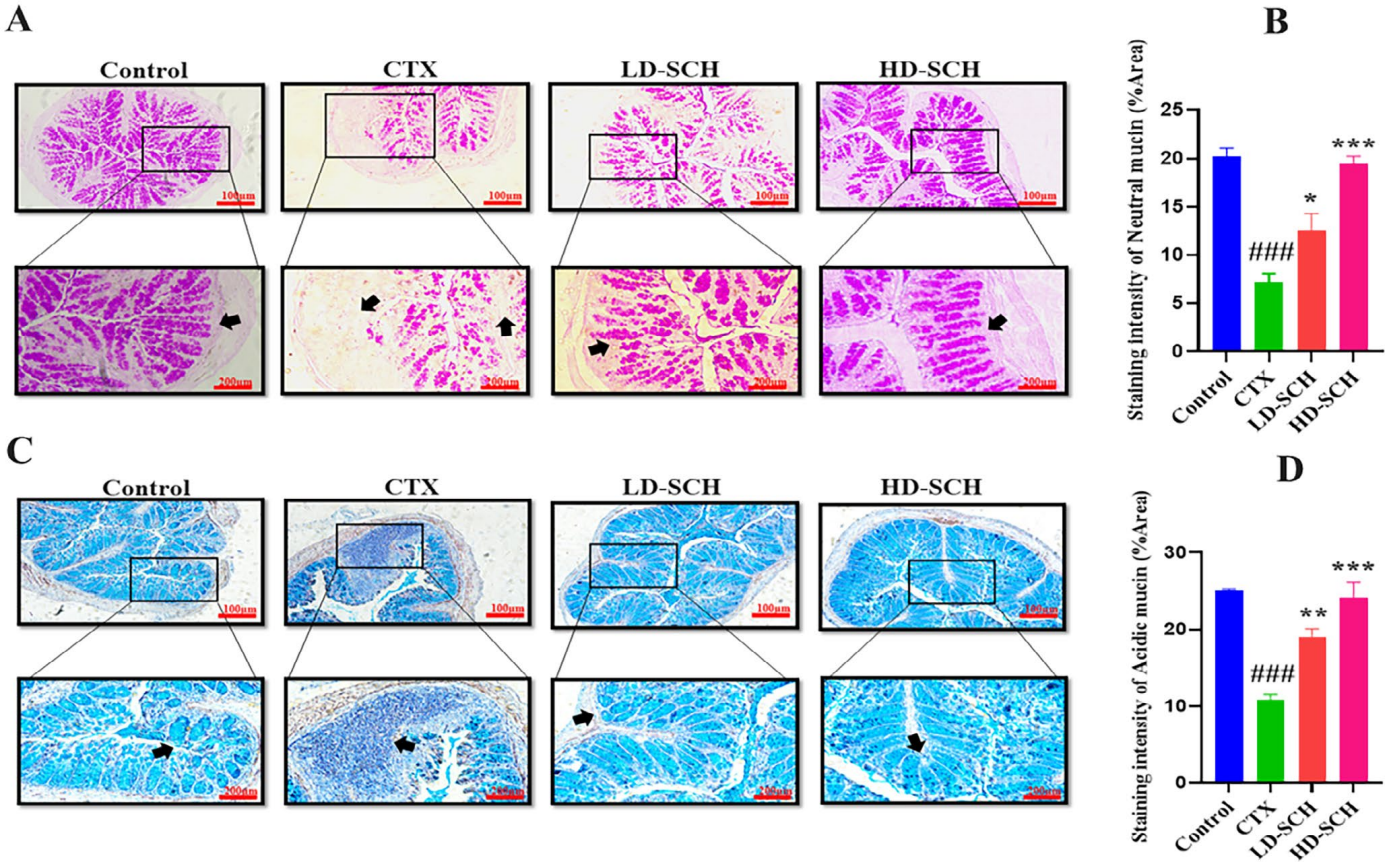
The SCH intake restored goblet cells. (A) Periodic acid staining illustrates the morphology of colon sections. The quantity of goblet cells generated was assessed in all groups. (B) Quantification Graph of PAS staining (C) Alcian blue staining of colonic tissue. The number of inflammatory cell infiltrates, as indicated by the red arrow, was assessed in all groups at both lower (20×) and upper (10×) magnification. (D) Quantification graph of AB staining, and results are shown as mean *±* SD. ^###^ *p* < 0.001 CTX *group*, *p < 0.05, **p < 0.01, ***p < 0.001, compared with the control group.

**FIGURE 9:**
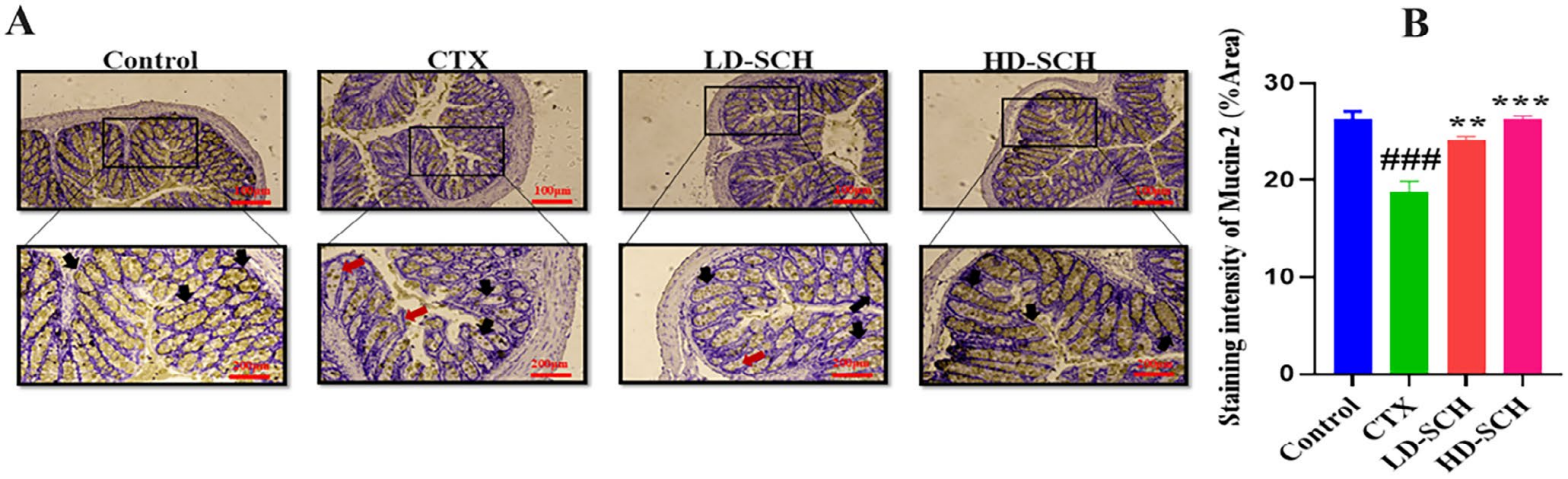
SCH treatment improves Mucin‐2 production in the colon. (A) Immunohistochemical staining of mucin expression in the colon of various groups. Mucin‐2 expression is indicated by a goldish color, the back arrow representing mucin expression and the red arrow representing inflammatory cells. Results magnification (upper 10×) (lower 20×), (B) Quantification graph of IHC staining (Mucin‐2), and results are shown as mean *±* SD. ^###^ *p* < 0.001 CTX group, **p < 0.01, ***p < 0.001, compared with the control group.

**FIGURE 10:**
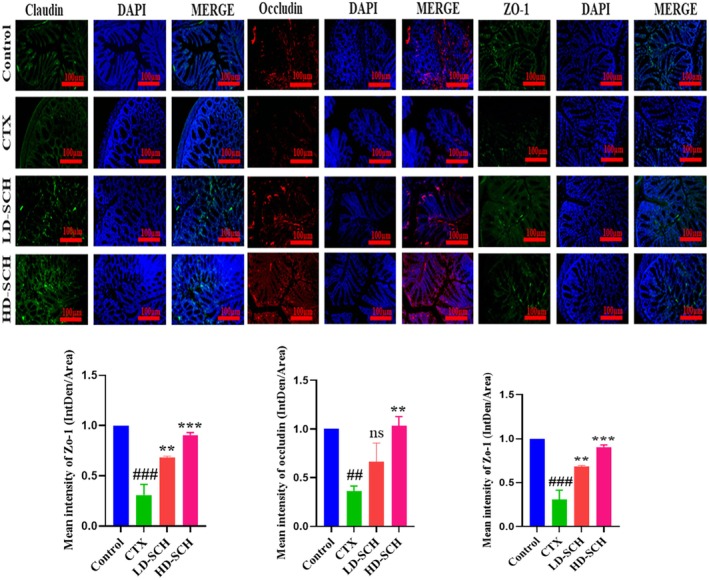
Immunofluorescent staining of tight junction protein expression, Claudin, Occludin, and ZO‐1 in the colonic epithelium. Original magnification: X20, scale bar: 100 μm, and the quantification graph of each immunofluorescent staining. Results: Ns (not significant) are shown as mean *±* SD. ^##^ *p* < 0.01, ^###^ *p* < 0.001 CTX group, **p < 0.01, ***p < 0.001, compared with the control group.

The CTX demonstrated a significantly decreased relative abundance of goblet cells compared to the control group. Periodic acid Schiff, Figure 8A, was utilized to stain neutral glycoproteins (purple‐stained) found in goblet cells of the colon. Alcian blue (AB) Figure 8C,D, staining demonstrated a substantial decrease in glycoprotein content in the CTX group compared to the control group, Figure 8A. Furthermore, SCH treatment enhanced and restored the substantial quantity of mucin expression and goblet cells in LD‐SCH and HD‐SCH treatment Figure 9A. Morphological alterations influence the tight junction protein expression in the colonic epithelium. To elucidate the effects of CTX on tight junction proteins, Mucin‐2, Claudin‐1, Occludin, and ZO‐1, and the mucosal barrier, which are among the most extensively researched, were examined. CTX administration decreased the expression of ZO‐1, Mucin‐2, Occludin, and Claudin‐1, whereas SCH treatment enhanced the extracellular barrier and elevated the expression of tight junction‐associated proteins, as illustrated in Figures 9 and 10.

### 3.11 SCH Restore Gut Microbiota Dysbiosis

**FIGURE 11:**
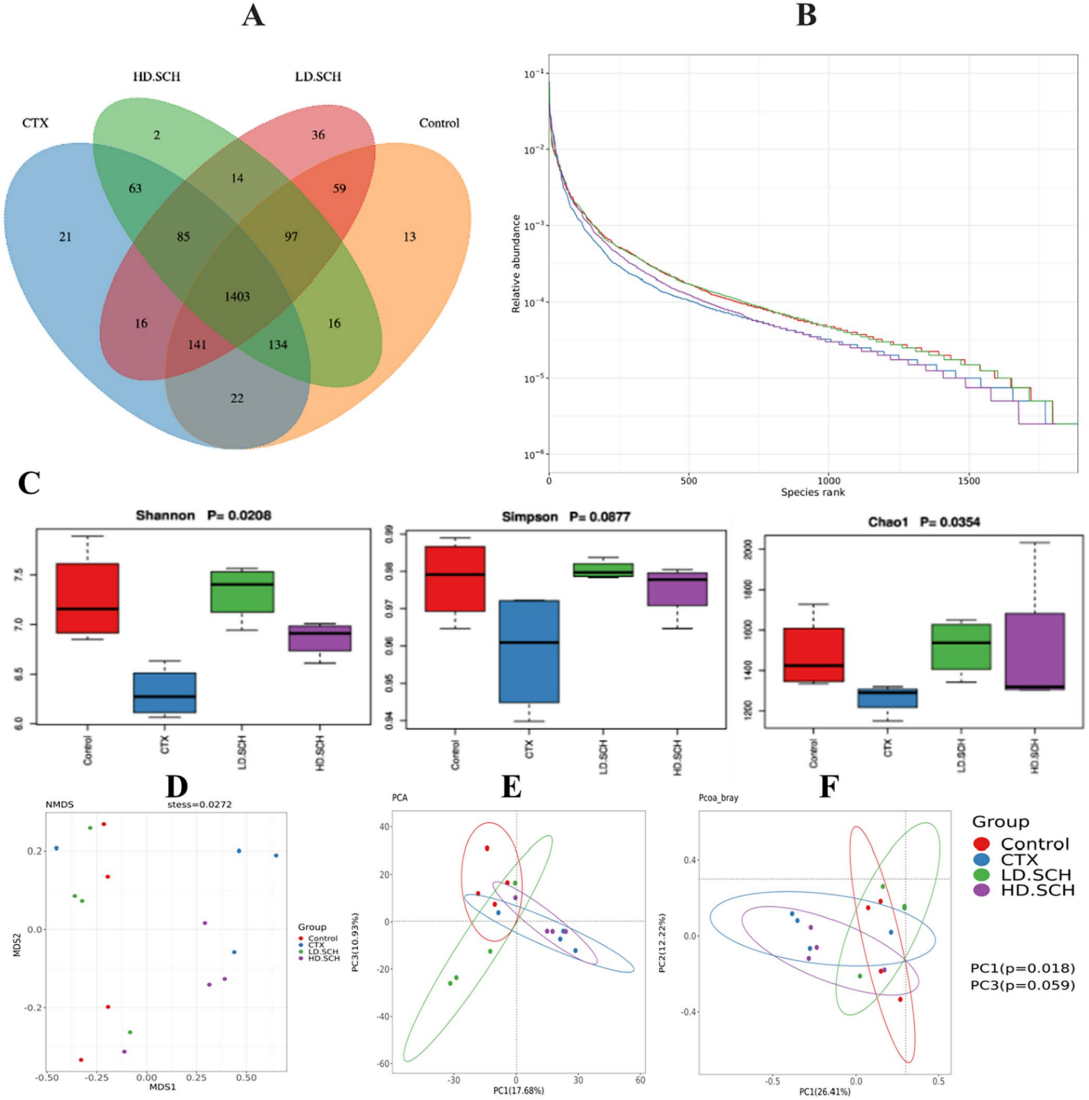
CTX produces disrupted microbiota by reducing the diversity of bacterial communities. (A) Venn diagram illustrates the bacterial OTUs among various LD‐SCH and HD‐SCH groups. (B) Rank abundance curve and illustrate both abundance and richness. (C) Alpha diversity measures, including Chao, Simpson, and Shannon, are utilized to assess richness, analogies, and discrepancies. (D–F) Illustrate the beta diversity index using NMDS, PCA, and PCoA plots. Each point represents a single sample and is distinguished by unique colors corresponding to various groupings.

The 16S rRNA gene was sequenced utilizing the Illumina NovaSeq 6000 to investigate the restorative impact of SCH on gut flora. The Venn diagram analysis revealed that all of the 1403 operational taxonomic units (OTUs) were expected for both the Control and experimental groups, facilitating the characterization of the overall structure, alterations, abundance, patterns, and diversity of various bacterial populations in the CTX and all SCH treatment groups. Furthermore, discrepancies among the groups were noted. The Control, LD‐SCH, and HD‐SCH groups demonstrated a significant increase in OTUs, but the CTX group revealed a decline in OTUs, as illustrated in Figure 11A.

The alpha diversity metrics were assessed utilizing the rank‐abundance curve, Shannon index, and observed species count. Rank abundance and refractive curves evaluated the bacterial richness and community richness among experimental groups and controls. In Figure 11B, each of the control and treatment groups displayed a distinct rank‐abundance curve. The curve's length and horizontal direction signify species richness and diversity. The control exhibits elevated species richness, which, overall, the SCH treatment groups succeeded in compared to the CTX groups. Alpha diversity indicates the variability and abundance of gut microorganisms. The data showed that SCH treatment augmented the alpha diversity indices relative to the CTX group. Additionally, beta diversity metrics were assessed by NMDS and principal component analysis (PCA) to elucidate the gut microbiome structure and evaluate the inter‐sample similarities and differences of species composition. Our findings indicated that each of the CTX samples was distinctly separated from the control group. Conversely, all dosage treatment groups in SCH, especially the medium and high doses, exhibited a tighter clustering compared to the control group Figure 11D–F. The examination of beta diversity indicates that CTX‐induced imbalance in the gut microbiota was significantly ameliorated following treatment with SCH.

**FIGURE 12:**
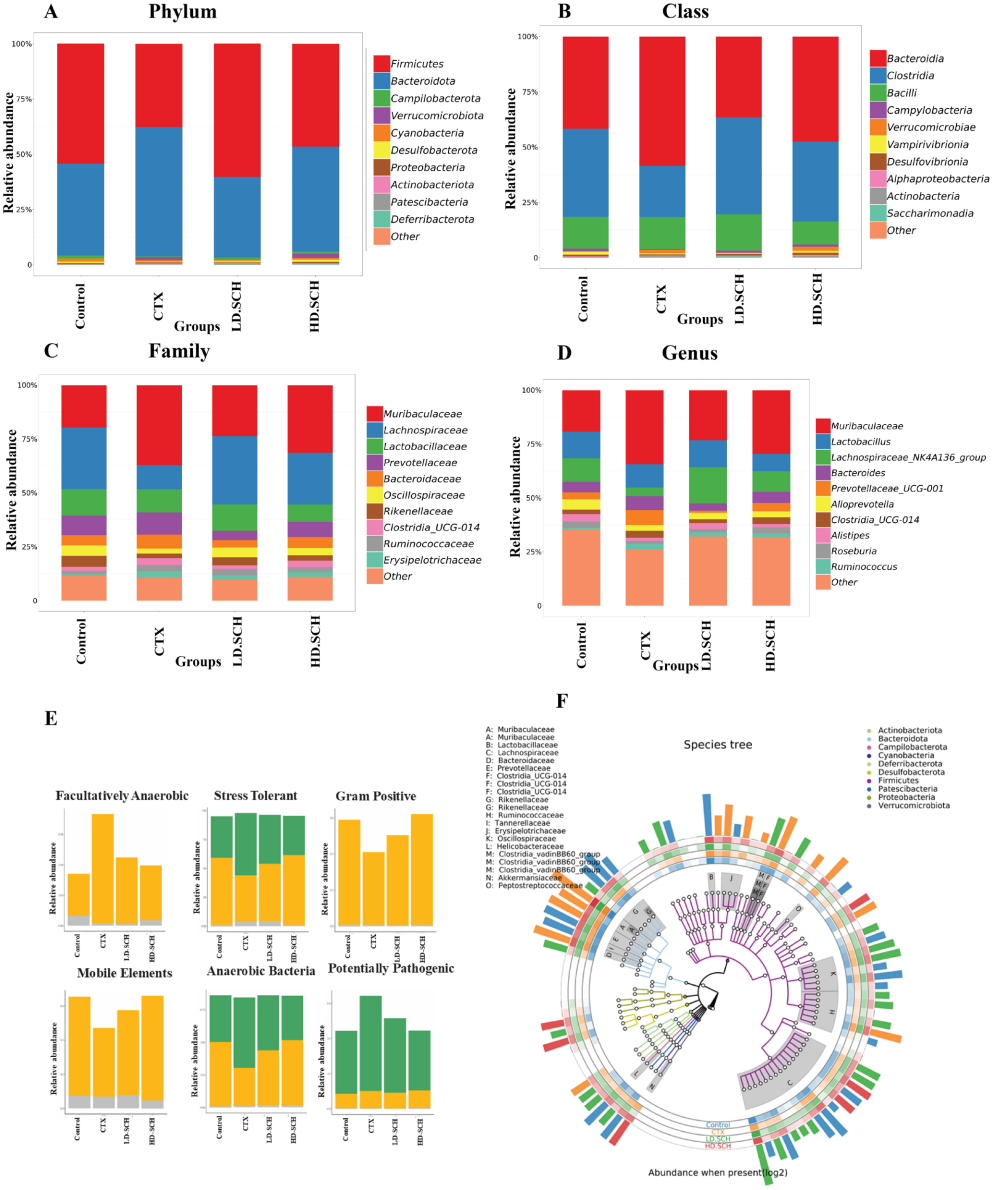
Microbial richness across several taxonomic levels and bug base analysis for distinct phenotypes. Bacterial enumeration across various taxonomic tiers: (A) phylum, (B) class, (C) family, (D) genus, (E) bug base analysis, (F) species phylogeny, corresponding to distinct phenotypes.

The taxonomic composition of the gut microbiota is analyzed at the class, phylum, genus, and family levels to determine the specific changes induced by SCH and CTX across all groups. *Firmicutes* and *Bacteroidetes* were the predominant bacterial phyla, comprising over 90% of the total bacterial population, as depicted in Figure 12A. Furthermore, it examines changes in microbial community organization at the class level. The results indicate that, relative to the control group, the CTX group exhibited an elevated abundance of *Verrucomicrobia* and *Bacteroidaceae* alongside a diminished abundance of *Firmicutes*, *Clostridia*, and *Campylobacterota*. HD‐SCH supplementation efficiently restores dysbiosis compared to the CTX group, as illustrated in Figure 12B. The abundance of fecal microbiota in the *Muribaculaceae* family is elevated relative to the CTX group, whereas *Lachnospiraceae* is diminished in the CTX group Figure 12C,D. Conversely, all HD‐SCH treatments reinstated all alterations. To assess prominent phenotypes across all experimental groups, bug base analysis revealed phenotypic distinctions linked to facultative anaerobes, anaerobic organisms, stress‐tolerant species, Gram‐positive bacteria, mobile genetic elements, anaerobic bacteria, and potentially dangerous bacteria Figure 12E. The results indicate that the microbiome phenotypes exhibit comparable relative abundance between the SCH treatment groups and the control group. Simultaneously, CTX groups exhibit significant variances. The functional genes encoding facultatively anaerobic, stress‐tolerant, Gram‐positive, and anaerobic bacteria were more prevalent in the CTX group. A species tree was created to depict the species diversity from the center circle to the exterior circle across various groups, as depicted in Figure 12F. LD‐SCH and HD‐SCH in both treatment groups efficiently reinstated the proportion of all afflicted phyla, underscoring SCH's therapeutic significance in rectifying gut dysbiosis (Table 4).

**TABLE 4:** Percentages of the top 10 bacterial phyla across various treatment groups.

	Control (%)	CTX (%)	LD‐SCH (%)	HD‐SCH (%)
*p_Firmicutes*	54.2	36.4	42.73	55.44
*p_Verrucomicrobia*.	17.0	19.0	18.3	16.9
*p_Bacteroidetes*	2.02	6.54	4.65	2.61
*p_Cyanobacteria*	0.05	0.07	0.43	0.12
*p_Deferribacteres*	0.12	0.05	0.06	0.02
*p_Tenericutes*	0.61	0.21	0.14	0.21
*p_Proteobacteria*	4.22	1.21	2.31	0.71
*p_Campylobacteria*	43.1	32.2	29.5	42.8
*p_Actinobacteria*	0.22	1.16	0.97	0.81
*p_TM7*	0.29	0.84	0.47	0.30

### 3.12 Correlation Between Gut Microbiota and Environmental Factors

The heatmap delineates the correlation patterns between various gut microbiota genera and different environmental or physiological factors. A color gradient from blue, indicating a negative correlation, to red, indicating a positive correlation, with statistical significance denoted by asterisks (* and **), (Figure S2). The data revealed two distinct microbial clusters with contrasting correlation patterns. The upper cluster, comprising genera such as *Turicibacter*, *Romboutsia, Faecalibaculum*, *Akkermansia*, *Bifidobacterium*, *Desulfovibrio*, and *Clostridium sensu stricto 1*, predominantly exhibited positive correlations (red) with TGF‐β, Thymus index, Colon index, and TNF‐α. This suggests that these bacteria may be associated with immunomodulatory functions, as TGF‐β and TNF‐α are cytokines that play crucial roles in immune regulation. Notably, *Clostridium sensu stricto 1* demonstrated strong positive correlations across multiple factors, particularly with immune‐related parameters. Conversely, the lower cluster, which includes genera such as *Alloprevotella, Lachnospiraceae* (*UCG‐006 and FCS020*), *Rikenellaceae*, *Helicobacter, Rikenella, Oscillibacter, Blautia, Roseburia*, and *UCG‐009*, generally shows negative correlations (blue) with these same factors. However, many of these genera correlated positively with IL‐4 and IL‐10, which are anti‐inflammatory cytokines, suggesting the potential anti‐inflammatory properties of these microbes. The spleen index parameter displayed mixed correlation patterns across both clusters, indicating a complex relationship between splenic function and gut microbiota composition. Water and food intake exhibited variable correlations, suggesting that dietary habits influence microbial communities differently across genera. The dendrograms on both axes indicate hierarchical clustering relationships, highlighting how certain bacteria and environmental factors cluster based on similarities in correlation patterns. The presence of statistically significant markers throughout the heatmap reinforces the robustness of these correlations, with some relationships reaching higher significance levels (** vs. *). This analysis provides valuable insights into how the gut microbiota interacts with host physiology and environmental factors, potentially influencing immune responses and metabolic functions, which have implications for understanding gut‐brain‐immune axis interactions in health and disease.

## 4 Discussion

The gut is the most significant system for digestion, absorption, and immunity. Immunity and gut microbiota constantly interact to improve immunological intestinal integrity and homeostasis (Mallet et al. 2014; Zhou et al. 2018). Peyer's patches, the first line of defense against damage, the lamina propria, and the epithelium make up gut immunological homeostasis. An imbalance in intestinal/gut homeostasis causes the gut microbial communities to become dysbiotic, which in turn causes several illnesses and aberrant immunological responses (Liu et al. 2018). A common anticancer medication and potent immunosuppressive agent, cyclophosphamide, can cause problems by disrupting the mucosal barrier and immune system, which decreases intestinal tight junctions and adherens junctions while possibly augmenting deleterious microbes (Li et al. 2017). CTX significantly influences various immune cells, affecting both the innate and adaptive immune systems. Administration of CTX alters the function and phenotype of dendritic cells (DCs). At high doses, CTX reduces the population of splenic CD103+ DCs and affects the antigen uptake and T cell priming capabilities of immature DCs. Furthermore, it induces changes in DC surface markers and enhances the expression levels of TLR, MyD88, and MAPK, which may influence Th cell polarization (Bao et al. 2020). CTX exerts a significant influence on T cell populations, particularly regulatory T cells (Tregs). The administration of low‐dose metronomic CTX results in a reduction of Tregs in both the bloodstream and tumor sites, while concurrently increasing the numbers of CD4+ and CD8+ T cells. This augmentation of immune function is observable in the circulatory and splenic regions, as well as within tumor environments (Zhong et al. 2020). Research has indicated that specific compounds, such as shrimp peptide hydrolysate, may play a role in reestablishing the balance of intestinal microbiota and enhancing immune function in individuals undergoing CTX treatment (Tang et al. 2022). The findings of this study align with previous research, demonstrating that the adverse effects of CTX on immune cell populations and gut microbiota highlight the importance of SCH in restoring immune balance and intestinal health. This is evidenced by SCH's ability to increase CD4+ and CD8+ T lymphocyte levels and to promote beneficial gut bacteria, such as Muribaculaceae.

However, peptide hydrolysates are recognized for their significant role in modulating the immune system, particularly in mitigating immune suppression induced by CTX. Studies suggest that hydrolysates obtained from sources such as shrimp, oyster, and sea cucumber can alleviate damage to immune organs, enhance cytokine production, and restore the intestinal lining in mice subjected to CTX treatment (Khan et al. 2022). For instance, the hydrolysate derived from the holothurian wall facilitated the release of pro‐inflammatory cytokines and contributed to the restoration of goblet cells and tight junction proteins in the colons of mice with compromised immune systems (Yan et al. 2023). Notably, the impact of peptide hydrolysates on the immune system extends beyond the mere activation of immune cells. These hydrolysates also influence the gut microbiota, which is integral to the regulation of the host's immune response. Numerous studies have demonstrated that hydrolysates can ameliorate CTX‐induced gut dysbiosis by promoting the proliferation of beneficial bacteria while inhibiting the growth of potentially harmful ones (Xiang et al. 2021).

For the activation of T‐cells, the presence of at least two signals is essential. Signal 1 is initiated when the T‐cell receptor (TCR)/CD3 complex recognizes major histocompatibility complex/peptide complexes on antigen‐presenting cells (APCs). Signal 2 is delivered through the costimulatory receptor CD28 (Dadwal et al. 2021). Interestingly, the interaction between T cells and APCs can lead to different outcomes depending on the specific molecules involved. For instance, B7‐H7 (HHLA2) has been found to inhibit T‐cell activation and proliferation when combined with TCR and CD28 stimulation, comparable to the inhibitory activity of PD‐L1 (Rieder et al. 2021). This suggests that some peptide hydrolysates could potentially modulate T‐cell responses through similar inhibitory pathways.

Therefore, in order to assess the gut microbiota, immunomodulation, and gut intestinal integrity, the effect of SCH on mice that were given CTX was examined in this study. Developed by enzymatically hydrolyzing scallop proteins, SCH is an abundant source of bioactive peptides with diverse health benefits and characteristics that have drawn interest due to its possible immunomodulatory effects (Kim and Mendis 2006). Factors that impacted the concentration of peptide extraction from marine resources included time, enzyme, temperature, solid–liquid ratio, and pH; for better quality of the product, the previously mentioned criteria were evaluated (Yu et al. 2018). Protein at 50°C with 1% papain enzyme is efficient, as seen by hourly increases in hydrolysate protein concentration. The MADIL‐TOF‐MS method investigated five low molecular weight peptides in the SCH extracts. Previous investigations have demonstrated immunomodulatory effects of peptide fractions < 6 kDa (Feng et al. 2012).

Immunomodulation is the term used to describe how substances that alter or reduce immune activity interact with the immune system, which comprises immunological organs, cells, and tissues (Gao et al. 2019). The spleen is the most substantial secondary lymphoid organ. It has a structure resembling lymph nodes, distinct compartments divided by connective tissue, and red and white pulp (Yu et al. 2018). The thymus and spleen, fundamental elements of the immune system, significantly contribute to both specific and nonspecific immunity (Chandrashekar and Venkatesh 2012). The differentiation, activation, and proliferation of immunological cells rise in correlation with the mass of the immunological organs and vice versa (Wang et al. 2016). Mice's body immune organ index dramatically decreased after receiving CTX. Still, all SCH treatment groups showed a significant increase (*p* < 0.05) in body weight, spleen, and thymus at post‐treatment compared to the model group. Peptide hydrolysate raises organ index and body weight, according to Wang's similar findings (Wang et al. 2010). There have been reports of CTX causing damage to the spleen, which is an essential organ in the immunological response (Okumura and Takeda 2017). According to the micromorphological observation, the spleens in the CTX‐induced group showed a disturbed structure, with an ambiguous boundary between the red and white pulp, an increase in reticular cells, and a drop in lymphocytes. These alterations result from immune organ atrophy and serve as a sign of immunosuppression. Following treatment with SCH, the margin of the pulps, the quantity of lymphocytes, and the spleen tissues were all regular.

The epithelial cells form the secondary defense of the intestinal barrier, directly aiding in the immunological upkeep of the gut. Epithelial cells perform a crucial function in pathogen response and in signaling the intestinal immune system through the secretion of inflammatory mediators and cytokines (Kuhn et al. 2014). Cytokines are diminutive hydrophilic glycoproteins produced by many cells, and cell division facilitates distinction and proliferation through plasma receptor molecules in contacting cells (Yu et al. 2018). Cytokines act as essential regulators and moderators of the immune response, with their release levels potentially reflecting the body's immunological activity. In CTX‐induced mice, a peptide derived from yak collagen hydrolysates markedly increased blood serum cytokine levels (IFN‐γ, TNF‐α, IL‐2), immunoglobulins (IgG, IgA), and upgraded both cellular and humoral immune responses. Mice treated with CTX exhibited a significant decrease in blood serum cytokines, consistent with previous findings (Hartmann and Meisel 2007). Following the administration of SCH, the expression of cytokines IL‐1β, IL‐4, TNF‐α, IL‐10, and immunoglobulins IgG and IgM was observed to be enhanced in our research. Prior research indicated that oyster peptide activated cytokines, including Interleukin 10 (IL‐10), Interleukin 4 (IL‐4), and Interleukin 2 (IL‐2) (Xiang et al. 2021), while Nibea japonica peptide markedly enhanced the cytokines production of such as IFN‐γ, TNF‐α IL‐2 (Yu et al. 2020). Numerous cells create tiny, soluble proteins known as cytokines, which communicate with various cells through plasma membrane receptors to promote cell division, differentiation, and proliferation. The majority of cytokine interactions cause target cells' gene expression to rise. These modifications in gene expression lead to the differentiation of B and T lymphocytes and the stimulation of cells such as macrophages (Hou et al. 2016). Activated Th2 cells produce IL‐4, which promotes B cell differentiation and proliferation and impacts mast cells, fibroblasts, endothelial cells, and macrophages (Abraha 2020). Th2 cells generate the anti‐inflammatory cytokine IL‐10, which stops Th1 cell growth. In particular, it prevents DCs and macrophages from promoting inflammation. Pro‐inflammatory cytokines like TNF‐α can alter the activity of T and B cells, among other immunocytes, potentially reversing some of the immunosuppressive effects of CTX (Terrando et al. 2010). The current study showed a significant increase in the mRNA expression of IL‐10 and IL‐4, and TNF‐α and TGF‐β were lower in the CTX‐induced group compared to the control group. Still, following SCH administration, cytokine levels of IL‐10 and IL‐4 were lower, and TGF‐β and TNF‐α were significantly higher, as was the case with TNF‐α, TGF‐β, IL‐4, and IL‐10 protein expression. This suggests that SCH enhances mouse immune function and lessens the intensity of immunosuppression by inhibiting CTX‐induced cytokine decrease.

In order to produce a strong immune response, which includes producing antibodies and activating cytotoxic T cells, CD4^+^ is necessary (Xiong and Bosselut 2012). Several cytokines that CD8^+^ T cells can produce strengthen the immune response and aid in the removal of infections (Clénet et al. 2017). The current findings indicate that the CTX condition resulted in a notable increase in the CD8^+^ T cell population and a substantial reduction in the frequency of splenic CD4+ T cells. Conversely, the quantities of CD8^+^ and CD4^+^ T cells in the spleen were markedly decreased in the HD‐SCH group. A notable augmentation in splenic CD8^+^ and CD4^+^ T cells and LPS‐induced lymphocyte proliferation was observed. A related study stated that there was a selective suppression of CD4^+^ T cell functions, but CD8^+^ T cell responses were mainly intact, based on related research impact of CTX therapy on the functional activities of splenic CD8^+^ and CD4^+^ T cells in a mouse model (Haley 2003).

Apoptosis is necessary for healthy lymphocyte and immunological function (Xu and Shi 2007). Overregulation of apoptosis will result in immunity‐related illnesses like cancer (Yu et al. 2018). Immunosuppression results from enhanced splenic lymphocyte death (Yu et al. 2018). The current findings demonstrated that whereas varying dosages of SCH decreased the apoptotic effects of CTX on lymphocytes, CTX increased splenic lymphocyte apoptosis in mice. These findings suggest that SCH may improve proliferation and suppress excessive splenic lymphocyte apoptosis, which could both promote organ damage and restore the immune system's cellular function.

Macrophages are essential immunocytes, playing an important role in innate host defense against pathogens and the removal of apoptotic and necrotic cells (Hou et al. 2012). The stimulation of macrophages was demonstrated by an increase in cellular size and the elongation of cytosolic projections. Auxiliary pathways in activated macrophages augment the immune system response (Hong et al. 2012). The impact of SCH dosing on morphological alterations in RAW 264.7 cells depended on concentration, leading to increased cell shape irregularity and augmented pseudopodia formation. Lactate dehydrogenase (LDH) resides in the cytosol of all healthy cells and cannot traverse the cell membrane at elevated concentrations unless membrane permeability is compromised (Arathi et al. 2022). It serves as a reliable diagnostic of cell membrane integrity (Zhang et al. 2019). The results demonstrated that SCH did not influence the integrity of macrophage membranes or the secretion of LDH at different dosages.

Intestinal epithelial cells maintain proper intestinal permeability, preserve structural integrity, and provide protection against pathogens and harmful substances (Lu et al. 2017). Tight junction proteins and goblet cells are crucial for maintaining intestinal architecture, ensuring barrier function, protecting the epithelial cell barrier, and regulating gut permeability. They secrete mucus, which effectively protects against invading pathogens and thus aids in the preservation of gut microbiome health (Maidana et al. 2016). Furthermore, goblet cells synthesize several components essential for maintaining the mucosal barrier and protecting against pathogens (Maidana et al. 2016; Okumura and Takeda 2017). Occludin and Claudin are integral membrane proteins that engage with the pericellular milieu and link neighboring cells. At the same time, ZO‐1 associates with claudin and occludin, potentially aiding in the maintenance of tight junction integrity (Buckley and Turner 2018). The staining outcomes in the CTX group demonstrated impairment of the intestinal and mucosal barriers, accompanied by a reduction of tight junctions and the protein of goblet cells. The SCH reinstates diminished mucosal permeability induced by CTX by replenishing goblet cell populations and enhancing the synthesis of proteins related to tight junctions. These results align with previous investigations utilizing cyclophosphamide (Ying et al. 2020; Xiang et al. 2021).

The gut microbiota profoundly influences the human immunological system. Gut bacteria are associated with physiological functions in the gastrointestinal tract and immunological tolerance. The result has fostered a new platform for characterizing and identifying gut microbiota and their association with various diseases (Gkouskou et al. 2014). Nutraceuticals enhance gut integrity and augment the host's functional activities, including absorption and immunological response (Garagnani et al. 2013). Feces contain the highest concentration of germs, along with the contents of the gastrointestinal tract, facilitating easy collection and minimizing disruption. They demonstrate changes in gut microbiota linked to health and disease (Xu and Zhang 2015). The composition of the gut microbiota was analyzed utilizing 16S rRNA from the Illumina NovaSeq 6000. The Chao1, Simpson, and Shannon indices were assessed to compare the quantity of OTUs in a sample; higher values signify more richness of advantageous gut microbiota. The Ace and Chao 1 indices were the principal measures for forecasting group abundance, while the Simpson and Shannon indices served as the leading indicators of group biodiversity (Yang et al. 2021). Alpha diversity diminishes microbiological richness and diversity following CTX treatments. In contrast, after SCH administration, the richness and diversity of the spices were both restored and augmented in the treatment group, as illustrated in Figure 11C. Beta diversity indices indicate that NMDS and PCA demonstrate significant variety among the groups. The analysis of beta diversity results reveals that the CTX group was markedly distinct from the control group, whereas the SCH treatment group showed closeness to the control group; CTX induces dysbiosis, but SCH promotes the restoration of microbiota dysbiosis. Prior research indicates that the abundance and variety of microbiota diminish following the treatment of CTX (Xu and Zhang 2015; Xiang et al. 2021). CTX has exhibited indiscriminate dysregulation of immune and intestinal mucosal cells, resulting in enteritis due to increased permeability, compromised intestinal barrier immunological function, and changes in the microbial populations of the small intestine (Caporaso et al. 2010; Chen et al. 2016). The gut microbiota composition was analyzed between the Control and CTX‐induced groups, demonstrating an increased prevalence of *Firmicutes* and a decreased prevalence of *Bacteroidetes* in the CTX group, consistent with previous studies (Gill et al. 2006; Caporaso et al. 2010; Ngo et al. 2012). Furthermore, dysbiosis of immunological function was seen in immunosuppressed mice, defined by an overabundance of B*acteroides* (Elinav et al. 2011). The fecal microbiota of mice subjected to chemotherapy exhibited an increase in Actinobacteria, Erysi, TM7, and Verrucomicrobia, while Campylobacteria and *Tenericutes* were diminished, similarly reported by Xu and Zhang (2015). The healthy gut microbiota of mice exhibits a well‐balanced mix of bacteria across various groups, correlating with low‐grade intestinal inflammation, irrespective of the mouse strain (Gu et al. 2013; Hildebrand et al. 2013). Our findings revealed links between CTX‐induced immune responses and variations in bacterial abundance among multiple bacterial groupings.

The Bugbase data reveal that microbial phenotypes in both the control group and the SCH treatment groups display comparable levels of phenotypes. The CTX‐induced groups exhibit significant variances. The functional genes associated with stress‐tolerant, anaerobic bacteria, and facultatively anaerobic bacteria were shown to be more prevalent in the CTX group. These findings align with previously published research by (Kanwal et al. 2020). Dysbiosis significantly influences the body's physiological functions and metabolic processes. Our study used the STAMP technique to assess the gut microbiome metabolome in mouse models. Our research suggested that SCH may improve energy utilization and nutrient absorption facilitated by CTX. We demonstrated that SCH improved carbohydrate metabolism while maintaining gut integrity, inhibiting pathogens, metabolites, intestinal flora composition, and remodeling gut metabolome functions in immunocompromised mice. This indicates that SCH may have an immunologically beneficial role.

The gastrointestinal tract plays a crucial role in digestion, absorption, and immune functions. However, our study has several significant limitations. Primarily, it employs a CTX‐induced immunosuppression model in mice, which may not accurately reflect the gastrointestinal physiology of humans. Although treatment with SCH demonstrated notable improvements in immune markers and gut microbiota, the study did not thoroughly explore the underlying mechanisms. Additionally, the methods used to assess gut microbiota composition, primarily 16S rRNA sequencing, may not have fully captured microbial diversity. Future research should incorporate metagenomic analyses to obtain a more comprehensive understanding. In conclusion, while the results suggest that SCH positively impacts immune function and gut microbiota, acknowledging these limitations is essential to guide future research.

## 5 Conclusion

The study emphasizes the crucial role of gut health in sustaining physiological processes and immunological responses, particularly concerning dysbiosis caused by CTX. Our research indicates that SCH significantly improves energy utilization, glucose metabolism, and nutrient absorption while maintaining gut integrity and fostering a healthy gut flora. Notably, the treatment with SCH not only alleviated the detrimental effects of CTX on immunological organ indices and lymphocyte apoptosis but also restored the equilibrium of gut microbiota diversity and abundance.

Moreover, SCH therapy enhanced cytokine profiles and increased CD8+ and CD4+ T cell lymphocyte numbers, indicating a significant immunomodulatory effect. These findings suggest that SCH holds promise as a potential therapeutic agent for protecting and restoring gut health and immune function in immunocompromised states.

In modern times, SCH is found in dietary supplements that help improve gut health and immunological function. These supplements are especially helpful for people going through chemotherapy or who have other diseases that cause dysbiosis. Functional foods that aim to support a healthy gut microbiome may also benefit from SCH.

To plan for the future, it is crucial to learn more about the bioactive peptides produced by SCH and how they work. Possible uses include creating targeted treatments for inflammatory bowel disease, autoimmune diseases, and other disorders that affect gut health. In conclusion, SCH's many advantages highlight its promise as a significant component of health promotion and disease prevention programs.

## Author Contributions

**Muhammad Ilyas:** conceptualization (equal), formal analysis (equal), validation (equal), writing – original draft (equal), writing – review and editing (equal). **Mujeeb Ur Rahman:** methodology (supporting), software (supporting). **Muhsin Ali:** formal analysis (supporting), software (supporting). **Ting Deng:** methodology (supporting), resources (supporting), software (supporting). **Nabeel Ahmed Farooqui:** methodology (supporting). **Sharafat Ali:** methodology (supporting). **Hidayat Ullah:** methodology (equal). **Yamina Alioui:** methodology (equal). **Renzhen Ma:** data curation (equal). **Shuming Lu:** funding acquisition (equal), project administration (equal), resources (equal), validation (equal). **Liang Wang:** formal analysis (equal), funding acquisition (equal), project administration (equal), resources (equal). **Yi Xin:** conceptualization (equal), project administration (equal), resources (equal), supervision (equal), validation (equal), writing – review and editing (equal).

## Conflicts of Interest

The authors declare no conflicts of interest.

## Supporting information


Figure S1

Figure S2

## Data Availability

The original data for this work are available upon email request to the corresponding author.
