# Supplementary figures and images for: Effect of Marine‐Derived Scallop Peptide Hydrolysate on Immune Modulation and Gut Microbiota Restoration in Cyclophosphamide‐Induced Immunosuppressed Mice
Source: Food Sci Nutr. 2025 Jul 23;13(7):e70421. doi: 10.1002/fsn3.70421 (PMC12284433; doi:10.1002/fsn3.70421)

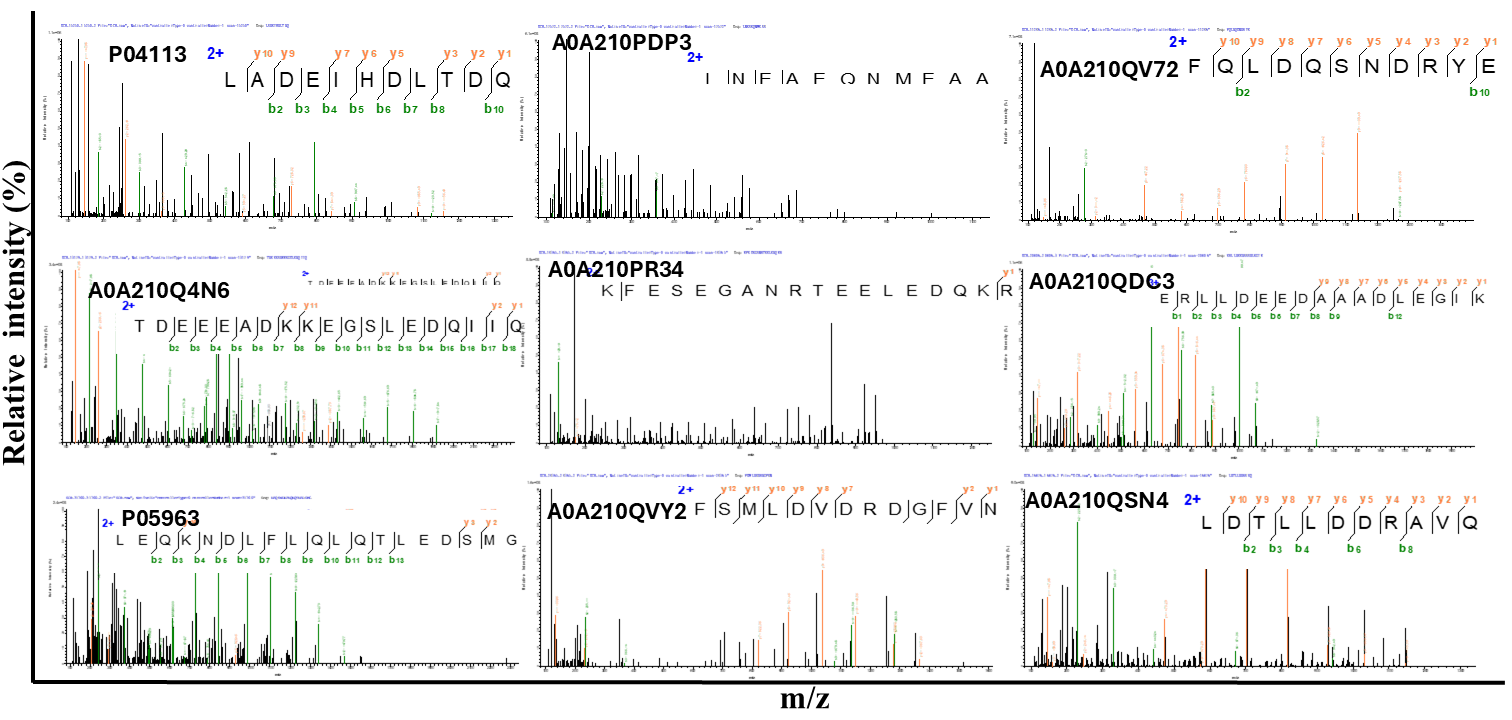

Supplement: Supplementary file 1 — Figure S1 [file FSN3-13-e70421-s002.tif]

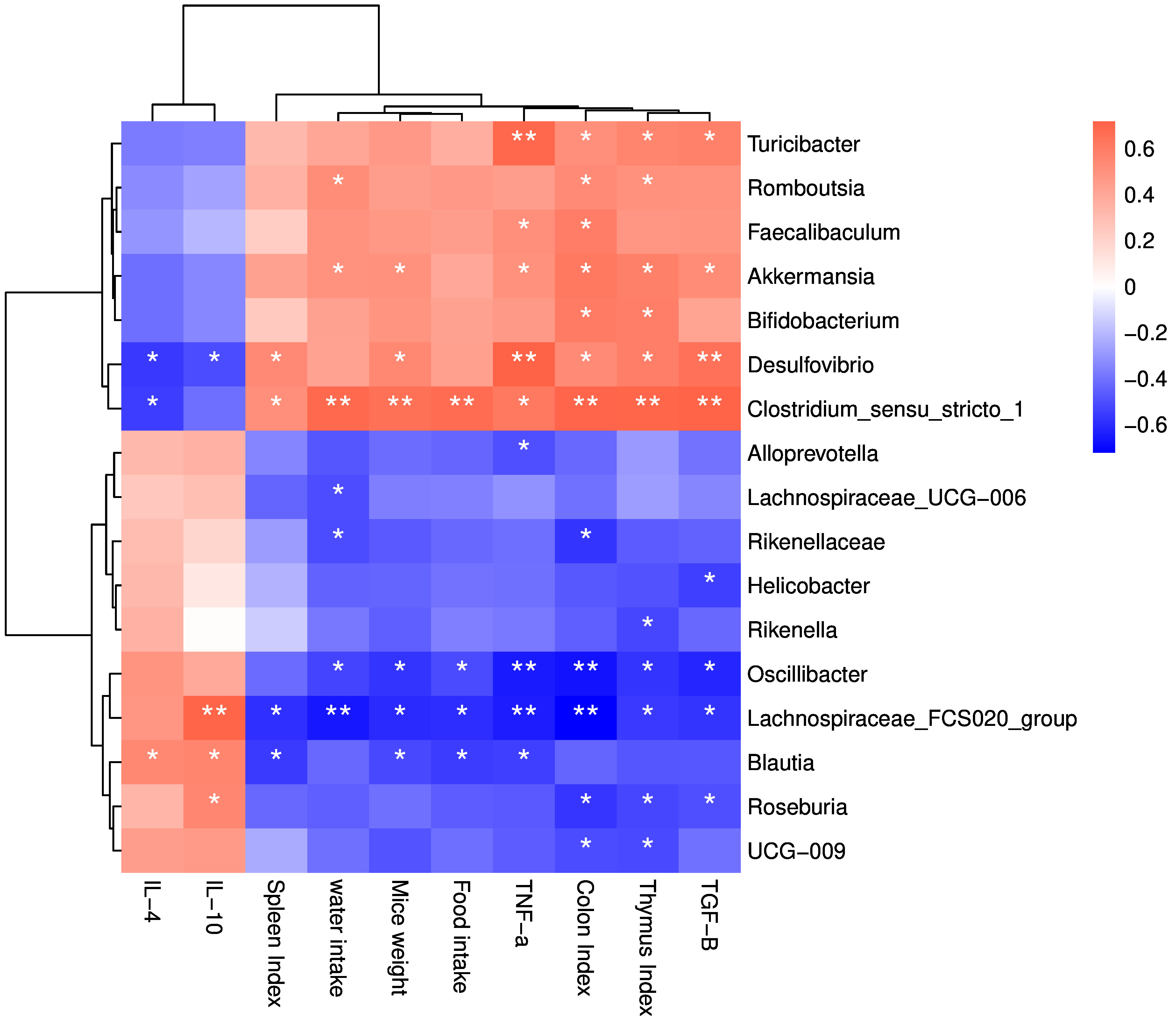

Supplement: Supplementary file 2 — Figure S2 [file FSN3-13-e70421-s001.jpg]
